# Integrated physiological and transcriptomic analysis reveals molecular distinctions between Huanglongbing- and Zn/Mn deficiency-induced chlorosis in citrus

**DOI:** 10.1186/s12870-026-09447-0

**Published:** 2026-08-08

**Authors:** Xiaoqing Cui, Sumiao Zhang, Lu Chen, Zheng Zheng, Xiaoling Deng, Xiaoling Deng, Wen Sang, Meirong Xu

**Affiliations:** 1 Guangdong Province Key Laboratory of Microbial Signals and Disease Control, Engineering Research Center of Biological Control, Ministry of Education/State Key Laboratory of Green Pesticide, College of Plant Protection, South China Agricultural University, Guangzhou, 515000 Guangdong China; 2 National Center for International Collaboration Research on Precision Agricultural Aviation Pesticide Spraying Technology, Guangdong Engineering Technology Research Center of Smart Agriculture, College of Artificial Intelligence, South China Agricultural University, Guangzhou, 510642 China

**Keywords:** Citrus, Greening, Mineral-deficient, Gene expression, Photosynthesis, Flavonoid, Starch, Phenylpropanoid

## Abstract

**Background:**

Citrus Huanglongbing (HLB) and zinc/manganese (Zn/Mn) deficiency induce similar chlorosis in young leaves, complicating field diagnosis. Despite visual similarities, the molecular mechanisms distinguishing these disorders remain unclear. This study employed integrated transcriptomic and physiological analyses to contrast HLB-specific responses with nutrient deficiency in *Citrus sinensis* (sweet orange) and *Citrus reticulata* 'Orah', aiming to identify the abiotic cause of chlorosis and clarify the underlying molecular divergence.

**Result:**

“*Candidatus* Liberibacter asiaticus” (CLas)-infected leaves showed significant downregulation of photosynthesis-related genes but upregulated starch/sucrose metabolism and phenylpropanoid biosynthesis, divergent from Zn/Mn-deficient leaves. Intriguingly, photosynthetic efficiency was independent of chlorosis severity. Starch accumulation was markedly higher and more consistent in HLB than in nutrient deficiency, serving as a predominant diagnostic marker. Flavonoid biosynthesis was significantly enhanced under both stresses, but the transcriptional induction was more pronounced and conserved across leaf positions in HLB. Additionally, plant-pathogen interaction pathways were activated exclusively in CLas-infected leaves, highlighting biotic stress-specific responses.

**Conclusion:**

We propose a practical diagnostic matrix—pairing starch assays with molecular signatures of biotic defense—to differentiate HLB from nutrient deficiency. Our comparative analysis reveals HLB establishes a unique "defense-priority" metabolic state, offering both actionable management tools and a novel conceptual framework for understanding its pathology.

**Supplementary Information:**

The online version contains supplementary material available at https://doi.org/10.1186/s12870-026-09447-0.

## Introduction

Citrus Huanglongbing (HLB), caused by the phloem-limited bacterium “*Candidatus* Liberibacter asiaticus” (CLas), is the most devastating citrus disease globally. One of its hallmark symptoms—zinc-deficient-like (ZDL) chlorosis in young leaves, characterized by green veins with interveinal yellowing—closely mimics the foliar symptoms of abiotic zinc (Zn) and manganese (Mn) deficiencies [1]. Under field conditions, especially in regions with alkaline, sandy, or heavily weathered soils where such deficiencies are prevalent [2, 3], this visual overlap severely complicates diagnosis based solely on leaf morphology [4, 5]. Differentiating these disorders is agronomically critical: HLB requires rapid tree removal to contain spread, whereas Zn/Mn deficiency can often be corrected through fertilization. Misdiagnosis thus carries significant economic and biosecurity risks.

Transcriptomic studies have elucidated how plants respond to these individual stresses. CLas infection suppresses photosynthesis-related genes and proteins, particularly in later stages, while upregulating starch synthesis and phenylpropanoid biosynthesis pathways [6, 7]. Early infection stages show minimal disruption to starch synthesis, but late-stage responses involve salicylic acid signaling and pathogenesis-related proteins [7]. Notably, redox and transport genes are upregulated in CLas-infected leaves, whereas immune receptor genes and light reaction-related pathways are down-regulated [8–10]. These findings highlight HLB’s systemic metabolic reprogramming, characterized by photosynthetic inhibition coupled with defense activation. In contrast, nutrient deficiencies trigger distinct transcriptional adjustments: Fe or Mn deficiency alters microtubule dynamics, phosphorylation states, and hormone signaling [11, 12], while boron deficiency induces lignin biosynthesis and chlorophyll degradation [5, 13]. Magnesium deficiency activates cell wall metabolism and reactive oxygen species pathways, exacerbating leaf vein lignification [14]. Critically, micronutrient supplementation (e.g., Zn, Mg) can mitigate HLB-induced starch dysregulation [15], suggesting overlapping yet divergent metabolic impacts. This partial overlap suggests that while molecular signatures exist, a systematic, side-by-side comparison under visually similar chlorosis is needed to identify which responses are specific, sustained, and pronounced enough to serve as reliable diagnostic markers.

The crosstalk between biotic and abiotic stress responses involves shared signaling components, including reactive oxygen species (ROS) and hormone pathways (e.g., salicylic acid, jasmonate, ethylene) [16]. However, these pathways exhibit stress-specific modulation: abiotic stress predominantly activates abscisic acid signaling, whereas biotic stress engages defense-related phenylpropanoid and plant-pathogen interaction pathways [17–19]. This mechanistic divergence hints at a potential molecular basis for distinguishing HLB from nutrient deficiency, but it has not been explicitly tested in a direct comparative framework where symptomology is held constant.

Therefore, to address this persistent diagnostic challenge, we conducted an integrated transcriptomic and physiological analysis directly comparing sweet orange (*Citrus sinensis*) and ‘Orah’ mandarin (*Citrus reticulata*) leaves exhibiting similar chlorosis symptoms caused either by CLas infection or by primary Zn/Mn deficiency. By focusing explicitly on this head-to-head comparison, our study aims to: (1) dissect the conserved and divergent molecular signatures between HLB- and deficiency-induced chlorosis, and (2) identify robust physiological and transcriptional markers that can underpin a more accurate, early-field diagnostic strategy.

## Results

### Phenotypic and nutrient profiling distinguishes symptomatic groups

**Fig. 1:**
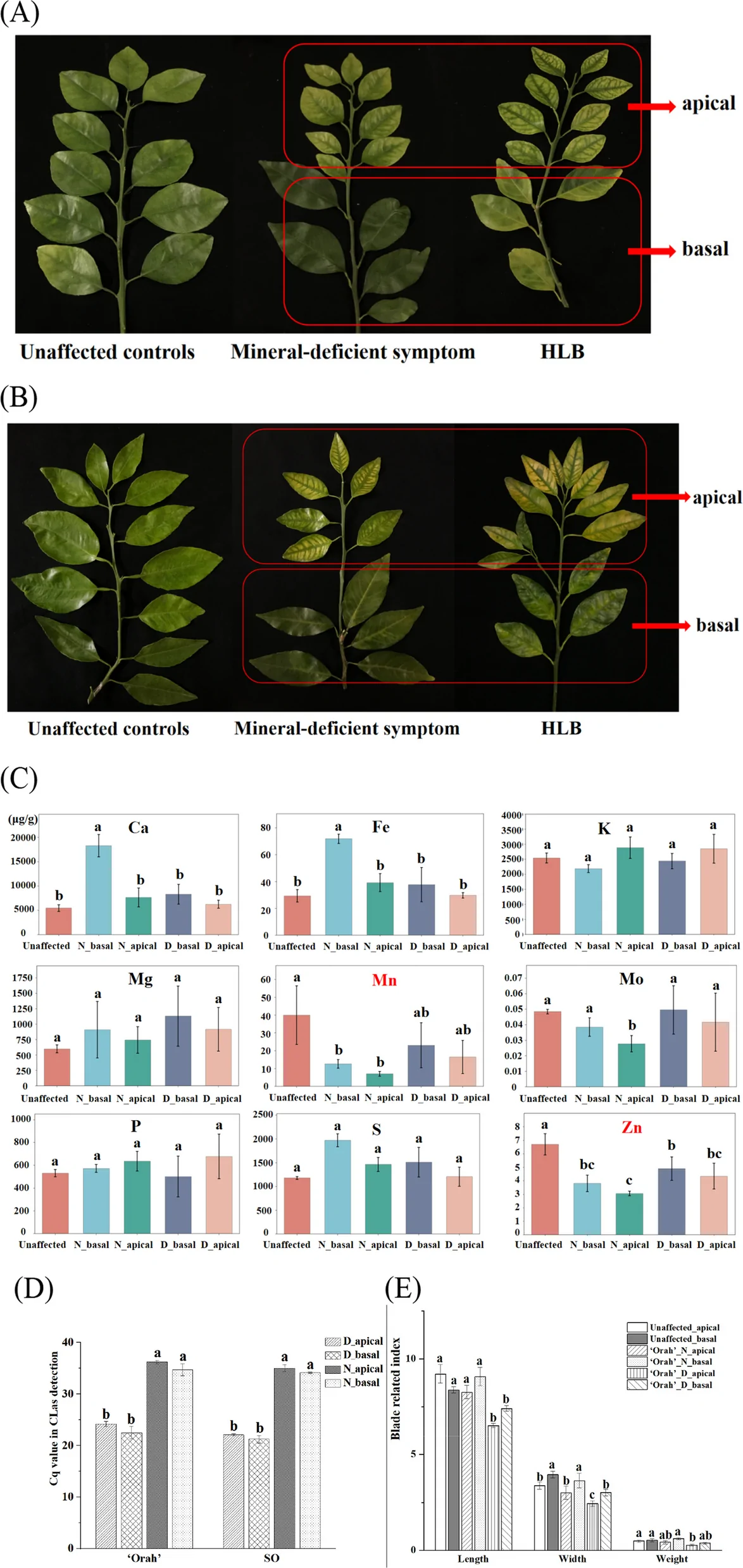
Symptoms, mineral nutrient contents (µg/g), and the Cq value of CLas in leaves of sweet orange and ‘Orah’ citrus. **A** Symptoms observed in sweet orange shoots. **B** Symptoms observed in ‘Orah’ shoots. **C** Mineral nutrient contents (µg/g) in lyophilized leaves of ‘Orah’. **D** The Cq value of CLas in leaves of sweet orange and ‘Orah’ citrus.** E** Leaf morphological indicators. SO, Sweet orange. **D **Huanglongbing-affected. N, Mineral nutrient-deficiency-affected. Apical Younger mature (upper) leaves. Basal Older mature (lower) leaves. All data are presented as means ± standard deviation of the mean (SD), error bars represent the SD. Bars with different letters indicate significant differences at the* p* < 0.05 level

In autumn and winter, the older leaves of CLas-infected sweet orange and 'Orah' exhibited typical blotchy mottling (BM) symptoms (Fig. 1A-B), while the younger leaves showed chlorotic patterns resembling ZDL. These ZDL symptoms could be easily misdiagnosed as nutrient deficiency-induced mottling. RT-qPCR analysis confirmed that the Cq value of CLas was significantly lower in HLB-affected leaves. Notably, no significant difference in the Cq value of CLas was observed between sweet orange and 'Orah' at different leaf ages on the same shoot, as shown in Fig. 1D. HLB significantly reduced the leaf biomass, whereas Zn/Mn-deficiency did not (Fig. 1E). Specifically, the leaf length (6.53 ± 0.54 mm) and width (2.44 ± 0.25 mm) of ZDL leaves, and the leaf length (7.42 ± 0.27 mm) of blotchy mottled older leaves on HLB branches, were significantly smaller than those of corresponding leaves on unaffected samples (*p* < 0.05). No differences in leaf dimensions were observed between unaffected and Zn/Mn-deficient ‘Orah’ shoots. ZDL leaves on HLB branches (0.27 ± 0.04 g) were lighter than unaffected leaves (0.53 ± 0.07 g, *p* = 0.027), but no weight difference were observed between the basal leaves on HLB branches and unaffected samples (0.48 ± 0.06 g, *p* = 0.402). Similarly, no significant weight differences were observed between unaffected and nutrient-deficient samples at corresponding positions.

Mineral nutrient analysis (iCAP TQ ICP-MS) revealed that the concentrations of Ca, Fe, K, Mg, Mo, P, and S in 'Orah' leaves remained consistent across leaf ages and chlorosis types (Fig. 1C). In contrast, Mn and Zn concentrations varied significantly among leaf types. Specifically, nutrition-deficient leaves had significantly lower Mn levels than the unaffected control (both apical and basal), whereas no significant difference was detected between unaffected leaves and HLB-affected leaves. Mn concentrations were also unaffected by leaf age within shoots. Unlike Mn, both nutrition-deficient and CLas-infected leaves showed significantly reduced Zn concentrations compared to unaffected leaves, with no age-dependent variation observed.

### Overview of transcriptomic data quality and differential expression

RNA sequencing of 24 samples generated high-quality data, with clean reads ranging from 5.93 to 6.75 Gb per sample. The base quality scores (Q20: 97.02—98.24%; Q30: 91.64—94.61%) and mapping rates (92.00% ± 1.16% to the reference genome) met the standards for reliable transcriptome analysis (Supplementary Tables S4, S5). Sample reproducibility was confirmed by Pearson correlation coefficients (> 0.906 between biological replicates; 0.918—0.971 within shoots) and PCA, where samples clustered by variety with 60% variance explained by PC1 and PC2 (Supplementary Figs. S1, S2).

**Fig. 2:**
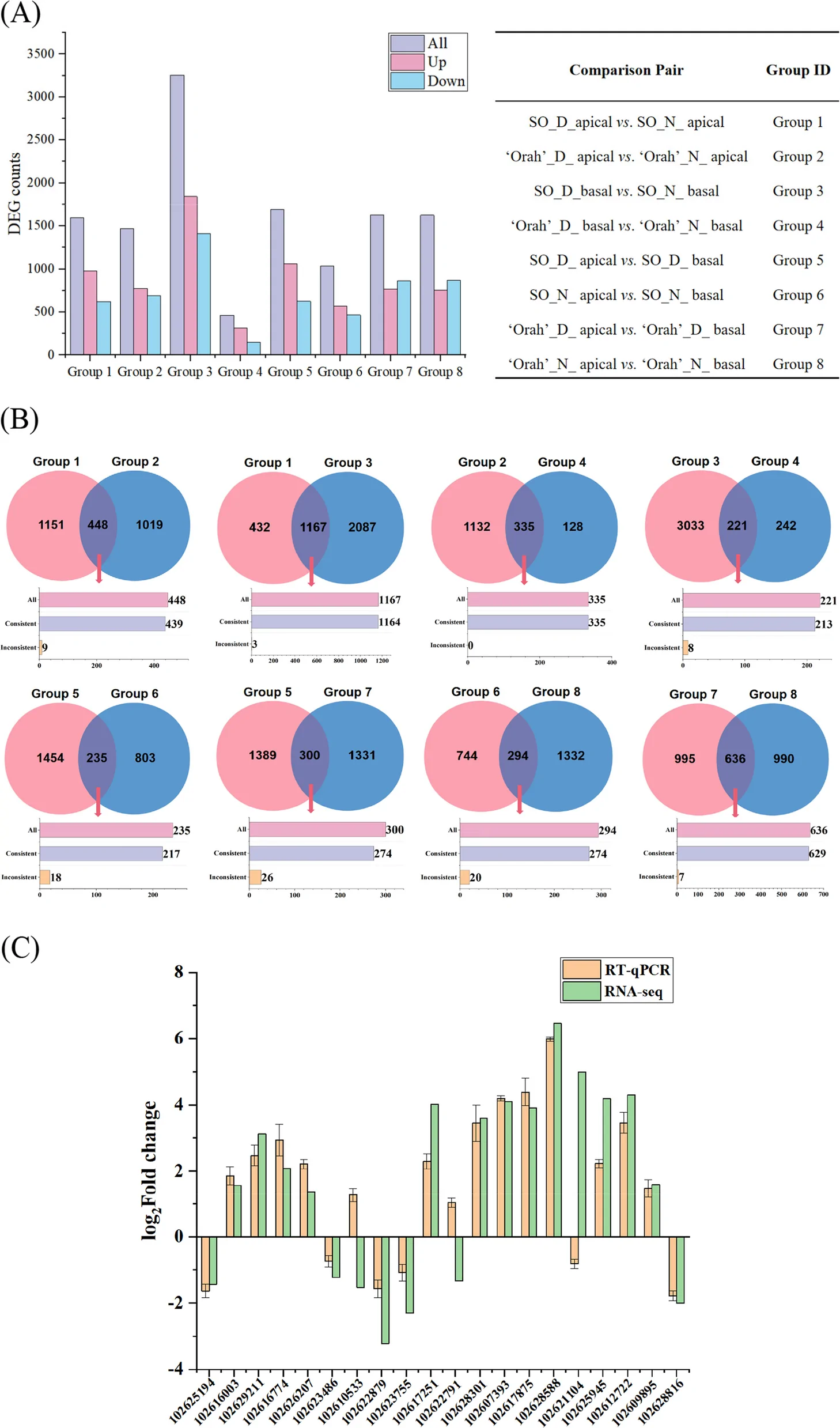
Differential expression analysis of genes across various comparison groups and validation of RNA-Seq data by qPCR. **A** Histogram depicting the number of differentially expressed genes (DEGs) in distinct comparison groups. **B** Venn diagrams illustrating the overlap and uniqueness of DEG counts among different comparison groups. **C** Histogram showing the gene expression levels (Log_2_fold change) of 20 selected DEGs, comparing RNA-Seq data with qPCR validation results. Detailed information regarding the comparison groups can be found in Table 1

Eight comparison groups were established to investigate the effects of stress type (HLB *vs*. nutrient deficiency) and leaf position (apical *vs*. basal) across sweet orange (SO) and 'Orah' mandarin. The detailed comparisons are outlined in Table 1. Group 3 (HLB *vs*. nutrition deficiency in basal leaves of SO, SO_D_basal *vs.* SO_N_basal) showed the most differentially expressed genes (DEGs) (n = 3254), indicating a particularly strong transcriptional reprogramming at this stage. Notably, six of the eight groups exhibited up-regulation-dominant DEG profiles (Fig. 2A). A substantial overlap of 1,167 DEGs was shared between Group 1 (SO_D_apical *vs.* SO_N_apical) and Group 3, with 1,164 (99.7%) of these showing consistent up- or down-regulation trends (e.g., 724 genes were up-regulated in both groups). Similarly, Group 2 ('Orah'_D_apical *vs.* 'Orah'_N_apical) and Group 4 ('Orah'_D_basal *vs.* 'Orah'_N_basal) shared 335 DEGs (Fig. 2B), all of which (100%) were consistently regulated. This high degree of conserved regulation across independent comparisons strongly suggests the activation of common, core pathway responses specific to HLB infection, distinct from those triggered by nutrient deficiency.

**Table 1:** Information of different comparison groups

Comparison Pair	Group ID	Biological Question
SO_D_apical *vs*. SO_N_ apical	Group 1	Stress Effect in Apical Leaves (HLB *vs.* Deficiency in sweet orange)
‘Orah’_D_ apical *vs*. ‘Orah’_N_ apical	Group 2	Stress Effect in Apical Leaves (HLB *vs.* Deficiency in 'Orah')
SO_D_basal *vs*. SO_N_ basal	Group 3	Stress Effect in Basal Leaves (HLB *vs.* Deficiency in sweet orange)
‘Orah’_D_ basal *vs*. ‘Orah’_N_ basal	Group 4	Stress Effect in Basal Leaves (HLB *vs.* Deficiency in 'Orah')
SO_D_ apical *vs.* SO_D_ basal	Group 5	Leaf Age Effect under HLB (Apical *vs.* Basal in Infected sweet orange)
SO_N_ apical *vs.* SO_N_ basal	Group 6	Leaf Age Effect under Deficiency (Apical *vs.* Basal in Deficient sweet orange)
‘Orah’_D_ apical *vs.* ‘Orah’_D_ basal	Group 7	Leaf Age Effect under HLB (Apical *vs.* Basal in Infected 'Orah')
‘Orah’_N_ apical *vs.* ‘Orah’_N_ basal	Group 8	Leaf Age Effect under Deficiency (Apical *vs.* Basal in Deficient 'Orah')

*SO* Sweet orange (*Citrus sinensis* (L.) Osbeck); 'Orah': *Citrus reticulata* 'Orah'; D: Huanglongbing-affected; N: Mineral nutrient-deficient; apical: younger mature leaves in a shoot; basal: older mature leaves in the shoot. E.g., 'SO_D_apical' denotes the apical leaves of CLas-infected sweet orange shoots

RT-qPCR validation of 20 selected genes confirmed RNA-seq accuracy (17/20 genes showed concordant expression trends, Fig. 2C), supporting the reliability of transcriptome findings.

### Comparative transcriptomics reveals distinct molecular signatures between HLB and nutrient deficiency

Although Zn/Mn-deficient and HLB-affected upper leaves ('up') exhibited visually similar chlorotic symptoms, RNA-seq uncovered distinct molecular responses. Group 1 (SO_D_apical *vs.* SO_N_apical) and Group 2 ('Orah'_D_apical *vs.* 'Orah'_N_apical) yielded 1599 and 1467 DEGs, respectively, with 448 shared DEGs (439 showing consistent regulation: 270 up-/169 down-regulated). Conversely, HLB unaffected old leaves in the Zn/Mn-deficient shoot (SO_N_basal, 'Orah'_N_basal) and HLB-affected (SO_D_basal, 'Orah'_D_basal) old leaves showed clear phenotypic divergence (green vs. blotchy mottling). Group 3 (SO_D_basal *vs.* SO_N_basal) and Group 4 ('Orah'_D_basal *vs.* 'Orah'_N_basal) generated 3254 and 463 DEGs, respectively, with 221 shared DEGs (213 consistently regulated: 184 up-/29 down-regulated). Notably, the higher DEG count in Group 3 (sweet orange) versus Group 4 ('Orah') suggests species-specific transcriptional responses to HLB in mature leaves.

**Fig. 3:**
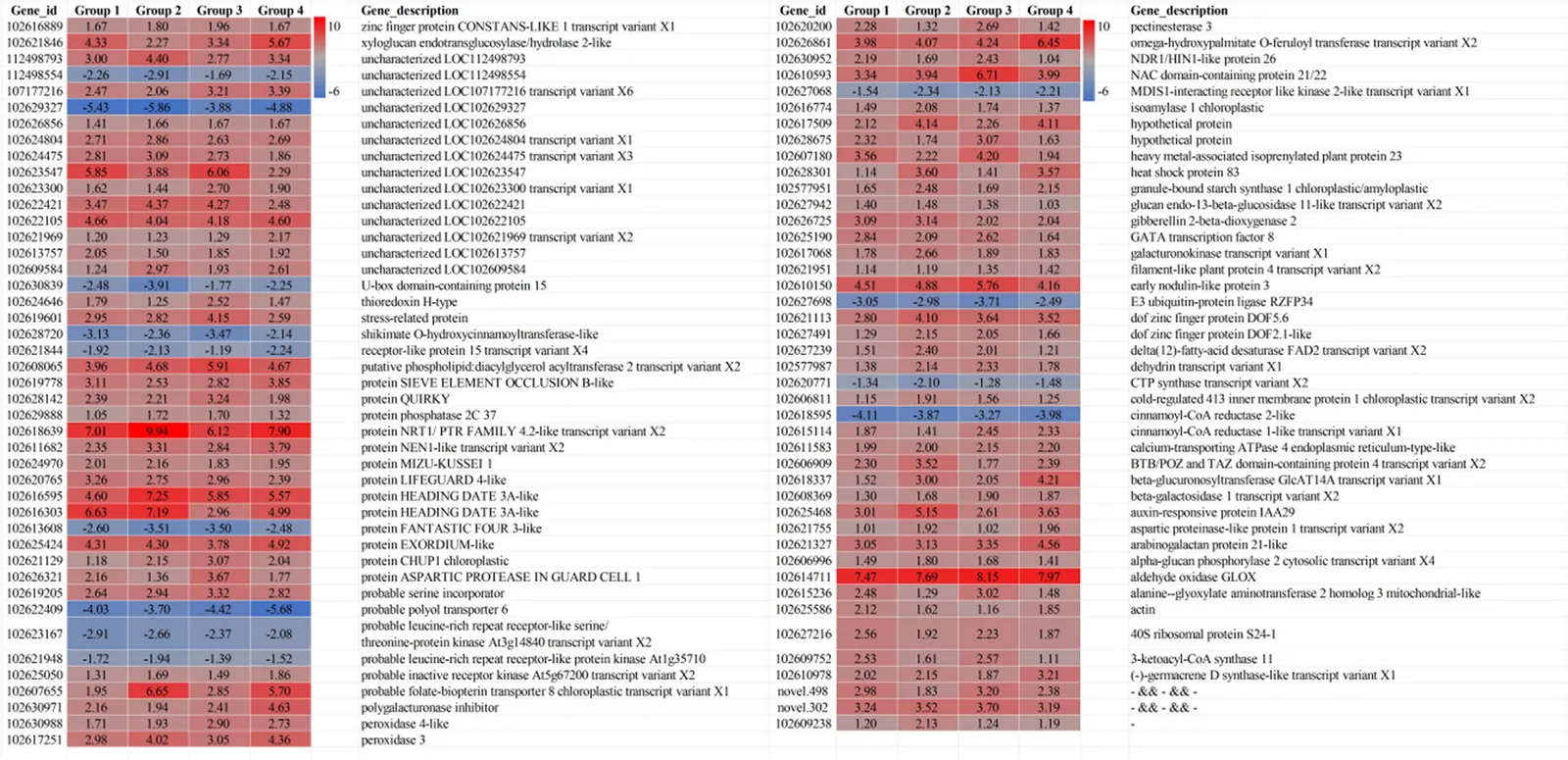
Log_2_fold change cluster diagram of common DEGs in four groups of samples related to ‘HLB *vs.* Zn/Mn-Deficiency’. Detailed information regarding the comparison groups can be found in Table 1

A total of 87 genes were identified as DEGs common to all four comparisons between HLB-infected and Zn/Mn-deficient leaves of the same variety and developmental stage (Group 1, Group 2, Group 3, and Group 4, Fig. 3). The expression trends of these 87 DEGs remained consistent within the four groups. Specifically, 13 genes were downregulated in HLB-affected shoots relative to Zn/Mn-deficient shoots, whereas the remaining 74 DEGs exhibited up-regulation. Notable among these genes are those encoding xyloglucan endotransglucosylase, polyol transporter, NAC domain-containing protein, cinnamoyl-CoA reductase, and aldehyde oxidase.

#### Photosynthesis suppression and metabolism alteration in HLB-affected ZDL leaves

**Fig. 4:**
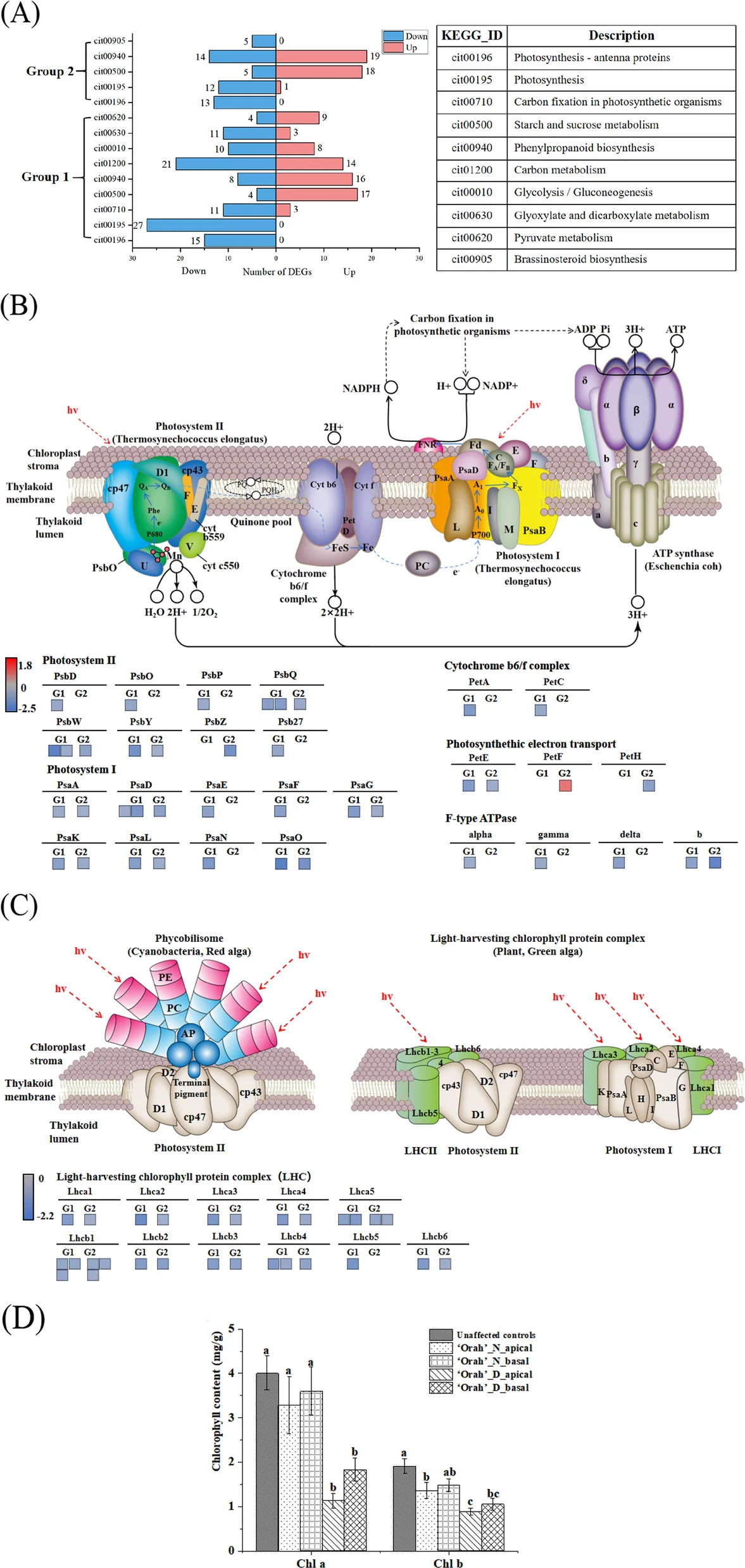
KEGG enrichment of photosynthesis pathways in upper leaves of sweet orange and ‘Orah’ shoots and physiological testing of the leaves on the ‘Orah’ shoots. **A** Histogram representing the KEGG enrichment analysis based on differentially expressed genes (DEGs). **B** KEGG pathway diagram depicting the photosynthesis process. **C** KEGG pathway diagram illustrating the photosynthesis-antenna proteins. **D** Chlorophyll concentration measurements. ‘Orah’ *Citrus reticulata* Blanco ‘Orah’. SO Sweet orange (*Citrus sinensis* (L.) Osbeck). **D** Huanglongbing-affected. N Mineral nutrient-deficiency-affected. Apical Younger mature leaves. Basal Older mature leaves. G1 Group 1, comparison between SO_D_apical and SO_N_apical. G2 Group 2, comparison between ‘Orah’_D_apical and ‘Orah’_N_apical. Chl a Chlorophyll a. Chl b Chlorophyll b. All data are presented as means ± standard deviation of the mean (SD), error bars represent the SD. Bars with different letters indicate significant differences at the *p* < 0.05 level

Comparative transcriptomic analysis of apical leaves (Group 1: sweet orange; Group 2: 'Orah') revealed distinct pathway alterations between HLB and Zn/Mn deficiency. In Group 1, nine pathways were significantly enriched, including three photosynthesis-related (cit00195, cit00196, cit00710), starch/sucrose metabolism (cit00500), and phenylpropanoid biosynthesis (cit00940) (Fig. 4A). In Group 2, there were four shared enrichment pathways (cit00195, cit00196, cit00500, cit00940) with Group 1 (Fig. 4A). Analysis of 448 co-DEGs identified cit00195, cit00196, and cit00500 as conserved responses across cultivars. 94.6% (53/56) and 96.2% (25/26) of photosynthesis-related DEGs were suppressed in Group 1 and 2, respectively. 23/25 down-regulated DEGs in Group 2 overlapped with Group 1, suggesting conserved photosynthetic inhibition in HLB. 81.0% (17/21) and 78.3% (18/23) of DEGs related to starch and sucrose metabolism were up-regulated in Group 1 and 2, indicating active carbohydrate accumulation. These findings suggest that photosynthesis, starch and sucrose metabolism, and phenylpropanoid biosynthesis pathways play crucial roles in the 'HLB *vs.* Zn/Mn-deficiency' comparison in the upper leaves of both cultivars. Comprehensive analysis of 59 photosynthesis-related DEGs from KEGG cit00195 and cit00196 revealed consistent downregulation of genes encoding: chlorophyll a-b binding proteins, PSI/PSII reaction center subunits, oxygen-evolving enhancers, ATP synthase subunits (Fig. 4B-C). Chlorophyll content is one of the most crucial factors determining the level of photosynthesis. Therefore, the chlorophyll content of the samples was determined (Fig. 4D). Chlorophyll content showed no significant difference between unaffected and Zn/Mn-deficient leaves across growth stages. CLas-infected leaves exhibited about 50% reduction in chlorophyll content compared to unaffected controls or Zn/Mn-deficient leaves (*p* < 0.01, Fig. 4D), corroborating transcriptomic data.

**Fig. 5:**
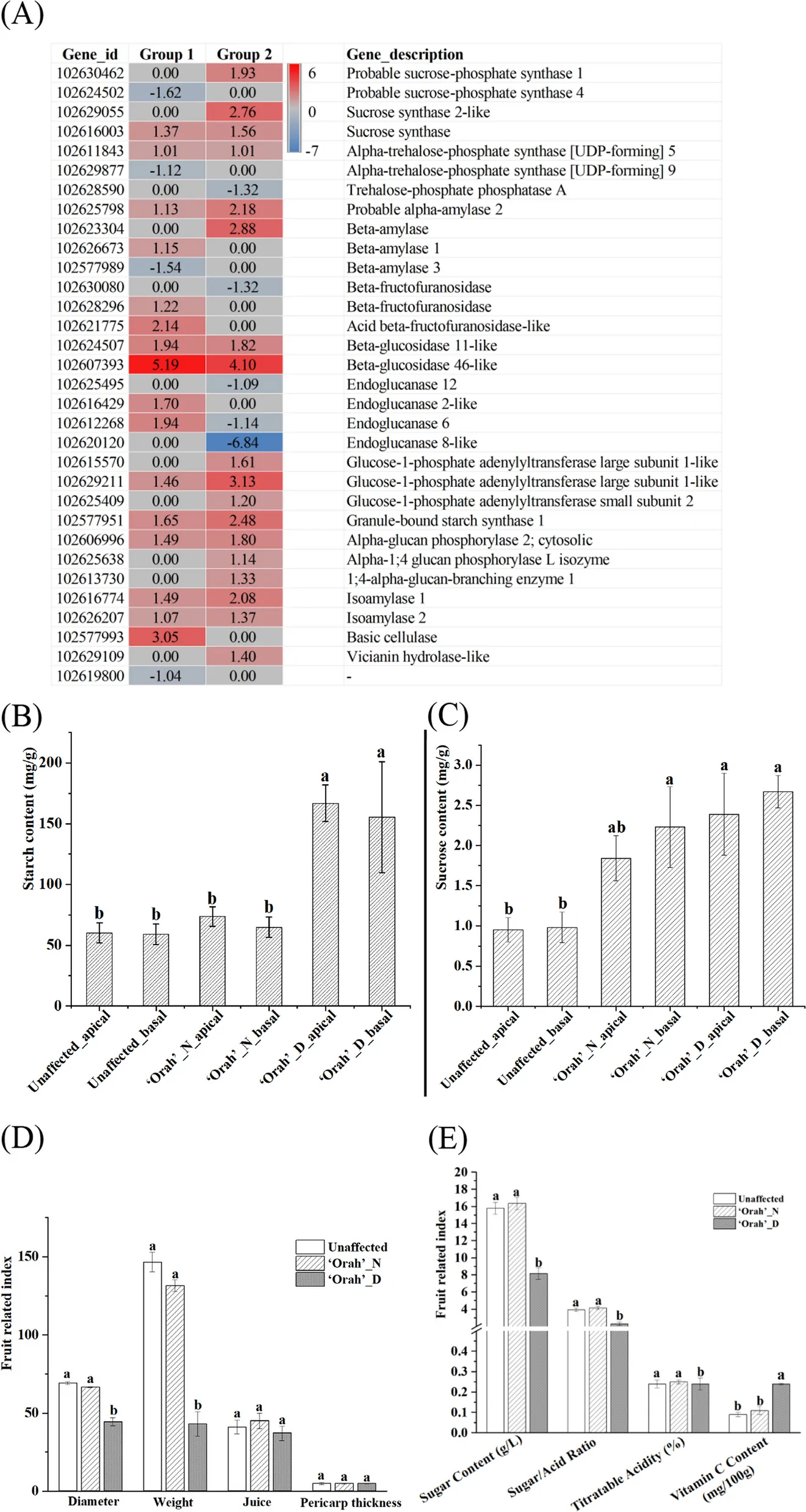
Log_2_fold change cluster diagram of the DEGs in starch and sucrose metabolism pathway, the starch and sucrose content in ‘Orah’ leaves and external and internal quality of ‘Orah’ fruits. **A** Represents the Log_2_fold change cluster diagram depicting DEGs involved in the starch and sucrose metabolism pathway. **B** Illustrates the starch content in ‘Orah’ leaves. **C** Displays the sucrose content in ‘Orah’ leaves. **D** External quality of ‘Orah’ fruits. **E** Internal quality of ‘Orah’ fruits. ‘Orah’ refers to *Citrus reticulata* Blanco ‘Orah’. SO denotes sweet orange (*Citrus sinensis* (L.) Osbeck). The abbreviations **D** and **N** are used to signify Huanglongbing-affected and Zn/Mn-deficiency-affected samples, respectively. Furthermore, ‘apical’ indicates younger mature (upper) leaves, while ‘basal’ represents older mature (lower) leaves. Group 1 SO_D_apical *vs.* SO_N_apical. Group 2 ‘Orah’_D_apical *vs.* ‘Orah’_N_apical. All data are presented as means ± standard deviation of the mean (SD), error bars represent the SD. Bars with different letters indicate significant differences at the *p* < 0.05 level

Transcriptomic and biochemical analyses revealed systematic changes in starch and sucrose metabolism. Thirty two DEGs involved in starch and sucrose metabolism (Fig. 5A), including sucrose-phosphate synthases (3), trehalose-phosphate synthases (3), granule-bound starch synthase 1 (1), beta-amylases (4), beta-fructofuranosidases (3), glucosidases (6), etc. We also found that the starch content in CLas-infected leaves was significantly higher than that in unaffected or Zn/Mn-deficient leaves, with no difference observed among different ages (Fig. 5B). Similarly, the sucrose content was significantly up-regulated in both HLB-affected leaves and nutrition-deficient basal leaves (Fig. 5C).

Given the profound metabolic dysregulation observed—particularly in carbon partitioning and sucrose trafficking—we hypothesized that these leaf-level disturbances would propagate to influence fruit development and final quality. To test this, we assessed a suite of fruit quality parameters. HLB negatively impacted 'Orah' fruit quality, reducing flavor and increasing vitamin C content, while Zn/Mn deficiency had minimal effects. HLB-affected fruits showed significant declines in external and internal quality, with significantly reduced size and weight, increased vitamin C content, and lower sugar content (8.17 ± 1.20 Brix) compared to unaffected (15.78 ± 1.19 Brix) and nutrient-deficient (16.38 ± 1.24 Brix) fruits (*p* < 0.05) (Fig. 5E). The sugar-to-acid ratio was also decreased only in HLB affected fruits. But peel thickness and juice yield remained unchanged (*p* > 0.05) (Fig. 5D-E). Unaffected and nutrient-deficient fruit samples showed no significant differences in quality indicators, indicating minimal impact of nutrient deficiency on ‘Orah’ fruit quality.

In the phenylpropanoid biosynthesis pathway, a total of 45 DEGs were implicated (Table S6). Anthranilate N-methyltransferases (6), caffeic acid O-methyltransferases (6), peroxidases (14) and shikimate O-hydroxycinnamoyltransferase (6) were noteworthy. Yet, no coherent regulation pattern emerged for these proteins as well. Intriguingly, in brassinosteroid biosynthesis pathway, all six DEGs related to cytochrome P450 were down-regulated in both Group 1 and Group 2, suggesting their potential as diagnostic biomarkers for HLB.

#### HLB-specific blotchy mottled symptom is molecularly associated with the pentose phosphate pathway and the plant-pathogen interaction pathway

**Fig. 6:**
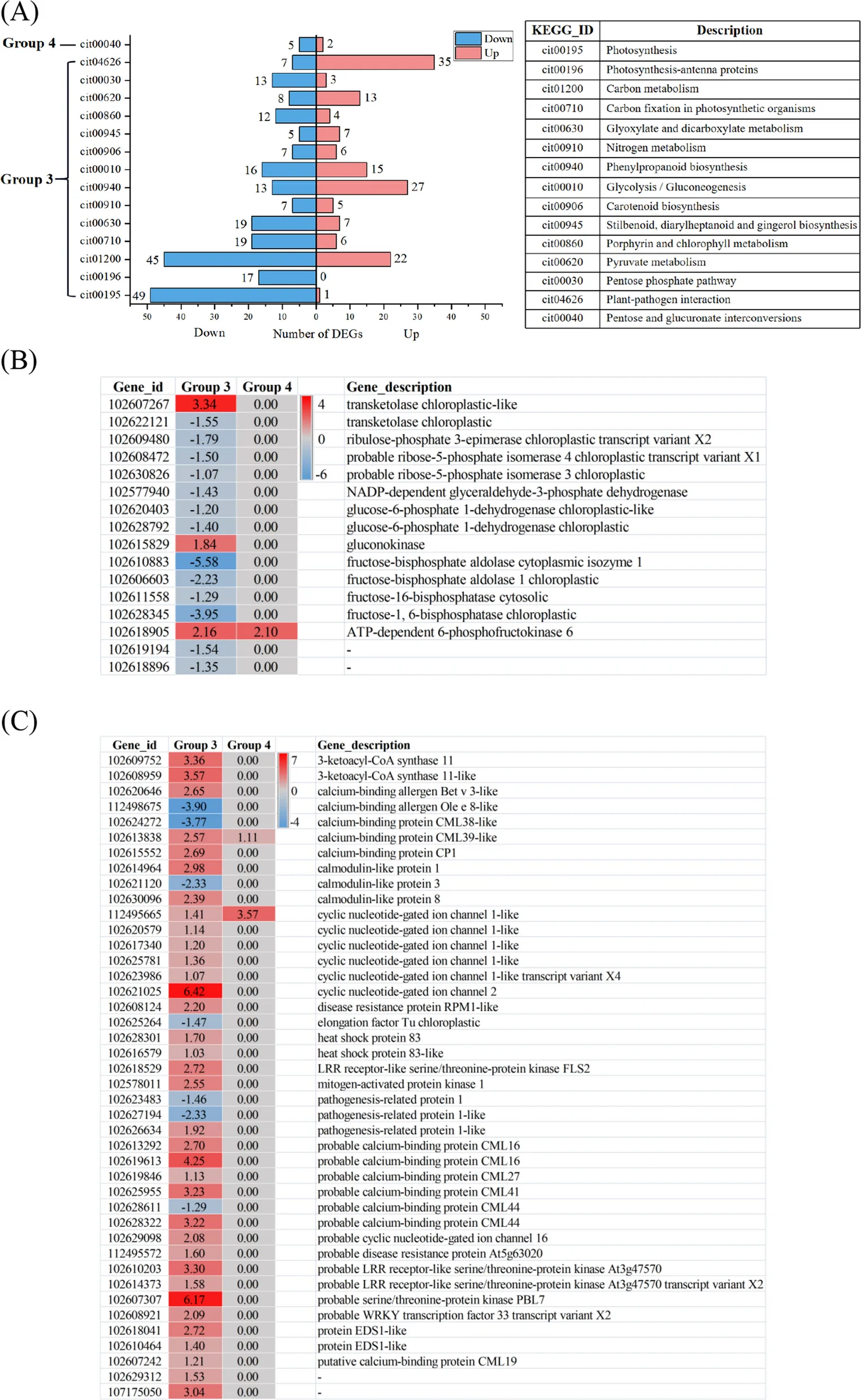
The histogram of KEGG enrichment analysis based on differentially expressed genes (DEGs) and Log_2_fold Change cluster diagrams of DEGs in the pentose phosphate pathway and plant-pathogen interaction pathway. **A** Histogram representing the KEGG enrichment analysis of DEGs in Group 3 and Group 4. **B** Log_2_fold change cluster diagram of the DEGs in the pentose phosphate pathway. **C** Log_2_fold change cluster diagram of the DEGs in the plant-pathogen interaction pathway. Group 3 SO_D_basal *vs.* SO_N_basal. Group 4 ‘Orah’_D_basal *vs.* ‘Orah’_N_basal

Analyzing the 3254 DEGs in Group 3 enriched 14 KEGG pathways (Fig. 6A). Overall, the photosynthesis process (cit00195 and cit00196) and the pentose phosphate pathway (cit00030) were suppressed, whereas the plant-pathogen interaction pathway (cit04626) was enhanced. Among 67 DEGs in photosynthesis, 66 were down-regulated. It is not surprising that "SO_N_basal" samples, lacking chlorotic symptoms, showed up-regulated photosynthesis-related genes compared to "SO_D_basal" samples. Specifically, 16 and 42 DEGs were linked to the pentose phosphate pathway and plant-pathogen interaction, respectively (Fig. 6B-C).

In the pentose phosphate pathway, up-regulated gluconokinase and transketolase genes, and down-regulated fructose-bisphosphate aldolase, glucose-6-phosphate 1-dehydrogenase, and fructose-1,6-bisphosphatase genes warrant further investigation. In the 42 DEGs associated with plant-pathogen interaction, two genes encoding 3-ketoacyl-CoA synthases, 12 calcium-binding protein genes, and six cyclic nucleotide-gated ion channel protein genes were significantly induced in CLas-infected sweet orange leaves. Notably, the genes induced in all four groups (102,613,838 and 112,495,665) will be further screened for potential biomarkers of HLB infection (Fig. 5B). Intriguingly, only one pathway (cit00040, pentose and glucuronate interconversions) was enriched in Group 4, involving seven DEGs, of which five were down-regulated. Unexpectedly, neither common KEGG pathways were enriched from both groups nor were they generated from the 221 common DEGs. This suggests that CLas induced pathway responses were more divergent between varieties in basal leaves, whereas apical leaves shared several enriched pathways.

### Leaf age modulates stress responses in a variety-specific manner

Differential expression analysis identified 1689, 1038, 1631, and 1626 DEGs in the comparisons of SO_D_apical *vs.* SO_D_basal (Group 5), SO_N_apical *vs.* SO_N_basal (Group 6), ‘Orah’_D_apical *vs.* ‘Orah’_D_basal (Group 7), and ‘Orah’_N_apical *vs.* ‘Orah’_N_basal (Group 8), respectively (Fig. 2A). Approximately half of the DEGs in each group exhibited either up-regulation or down-regulation. Sweet orange comparisons yielded 235 common DEGs, while 'Orah' comparisons had 636 (Fig. 2B). Among these 636 DEGs, 629 DEGs (98.90%) were consistently up- (314) or down-regulated (315) in both 'Orah' groups. Similarly, 217 of the 235 DEGs in the sweet orange groups were either up-regulated (121) or down-regulated (96) in both groups. These results suggest that the following conclusions drawn from these data are reliable.

**Fig. 7:**
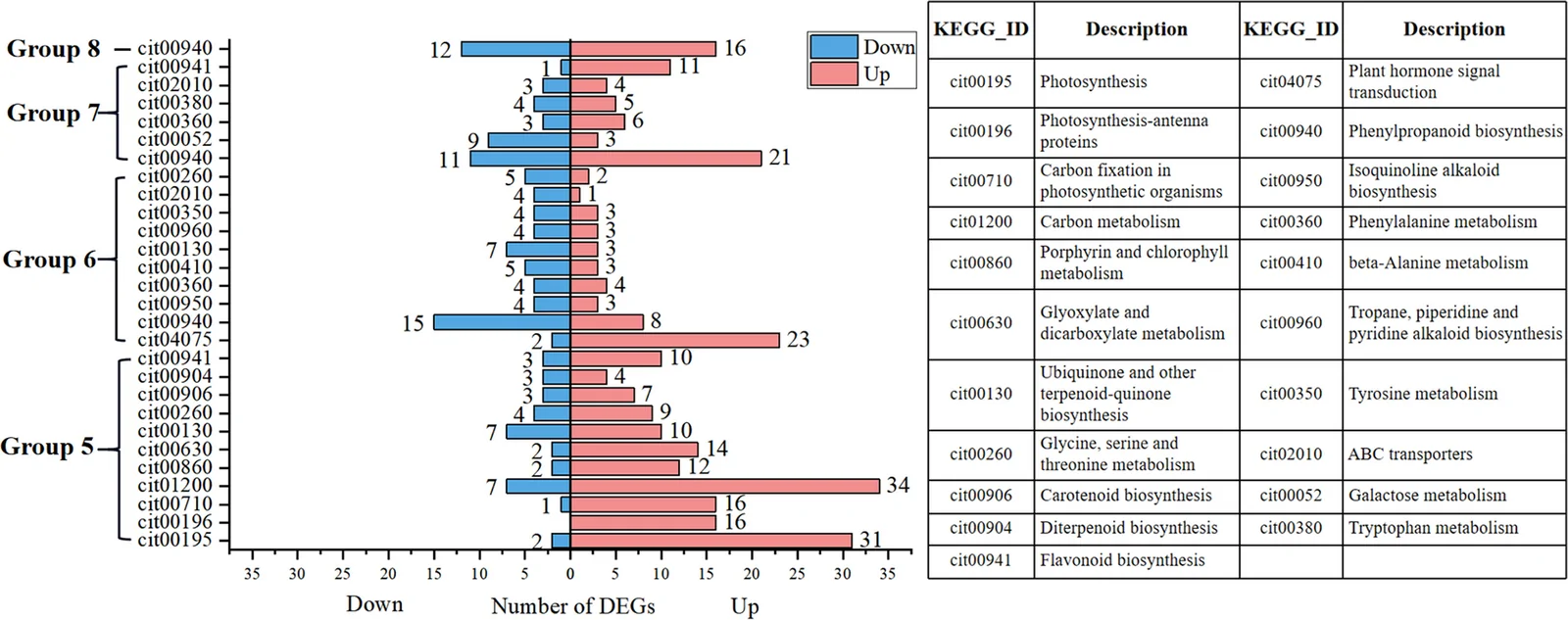
Histogram representing KEGG enrichment analysis of DEGs related to leaf age in sweet orange and 'Orah'. Detailed information regarding the comparison groups can be found in Table 1

Eleven, ten, six, and one KEGG pathways were enriched in Groups 5 through 8, respectively (Fig. 7). The pathways for ubiquinone and other terpenoid-quinone biosynthesis (cit00130) and glycine, serine, and threonine metabolism (cit00260) were significantly enriched in Groups 5 and 6. The flavonoid biosynthesis pathway (cit00941) was significantly enriched in Groups 5 and 7. The phenylpropanoid biosynthesis pathway (cit00940) was significantly enriched in Groups 6, 7, and 8. The ABC transporters (cit02010) and phenylalanine metabolism (cit00360) pathways were significantly enriched in Groups 6 and 7. KEGG enrichment analysis of the 217 DEGs with the same trend in Groups 5 and 6 revealed three significant enrichment pathways: phenylalanine metabolism (cit00360), ubiquinone and other terpenoid-quinone biosynthesis (cit00130), and isoquinoline alkaloid biosynthesis (cit00950). However, no significant enriched pathway was identified from the 629 common DEGs in Groups and 7 and 8.

#### Carbon metabolism and photosynthesis pathways are induced in ZDL leaves compared to BM leaves in HLB-affected sweet orange

After HLB affection, sweet orange leaves show chlorosis in winter. The apical HLB-affected leaves are ZDL, while mature basal leaves are BM. In Group 5 (SO_D_apical *vs.* SO_D_basal), a total of 73 DEGs (with 64 up-regulated) were involved in the photosynthesis-related pathways (Table S7). Seventeen DEGs (16 up-regulated) and 41 DEGs (34 up-regulated) contributed to carbon fixation in photosynthetic organisms (cit00710) and carbon metabolism (cit01200), respectively (Table S8). The majority of the 73 and 58 proteins were presumed to be located in the chloroplast. In summary, compared to BM leaves, the carbon metabolism and photosynthesis pathways in the chloroplast were activated in ZDL leaves.

Specifically, all 16 DEGs encoding chlorophyll a-b binding proteins in chloroplasts were upregulated in the ZDL younger leaves versus blotchy mature leaves, with Log_2_fold change values from 0.78 to 2.02. Another 16 DEGs related to photosystem I (PSI) or PSII reaction center subunits/proteins were all upregulated (Log_2_FC = 1.11–2.01) in Group 5. These DEGs encoding oxygen-evolving enhancer proteins, ATP synthase subunits, magnesium-chelatases, or magnesium protoporphyrin IX methyltransferase were proposed to contribute to enhanced photosynthesis. Intriguingly, despite ZDL leaves appearing more yellow than BM leaves (Fig. 1A), transcriptome analysis showed higher photosynthesis levels in HLB-affected ZDL leaves than in HLB-induced blotchy leaves.

Regarding the carbon metabolism pathway, DEGs encoding alcohol dehydrogenase-like proteins, cysteine synthase-like proteins, fructose-1,6-bisphosphatase, glyceraldehyde-3-phosphate dehydrogenases, glycine dehydrogenase, malate dehydrogenases, etc. were upregulated. A specific DEG (102,621,953) annotated as alcohol dehydrogenase-like 2 was highly expressed in the ZDL leaves, with a Log_2_fold change of 3.76. Moreover, DEGs encoding a ribulose bisphosphate carboxylase small chain, a phosphoenolpyruvate carboxykinase (ATP)-like protein, and a sedoheptulose-1,7-bisphosphatase were also significantly induced. Collectively, the carbon metabolism in the HLB-affected ZDL leaves of sweet orange was more robust than in the BM leaves. Unlike sweet orange, ‘Orah’ did not show strong age-related induction of carbon metabolism genes, indicating variety-specific acclimation.

#### Negative association between flavonoid biosynthesis pathway and leaf age under HLB stress

**Fig. 8:**
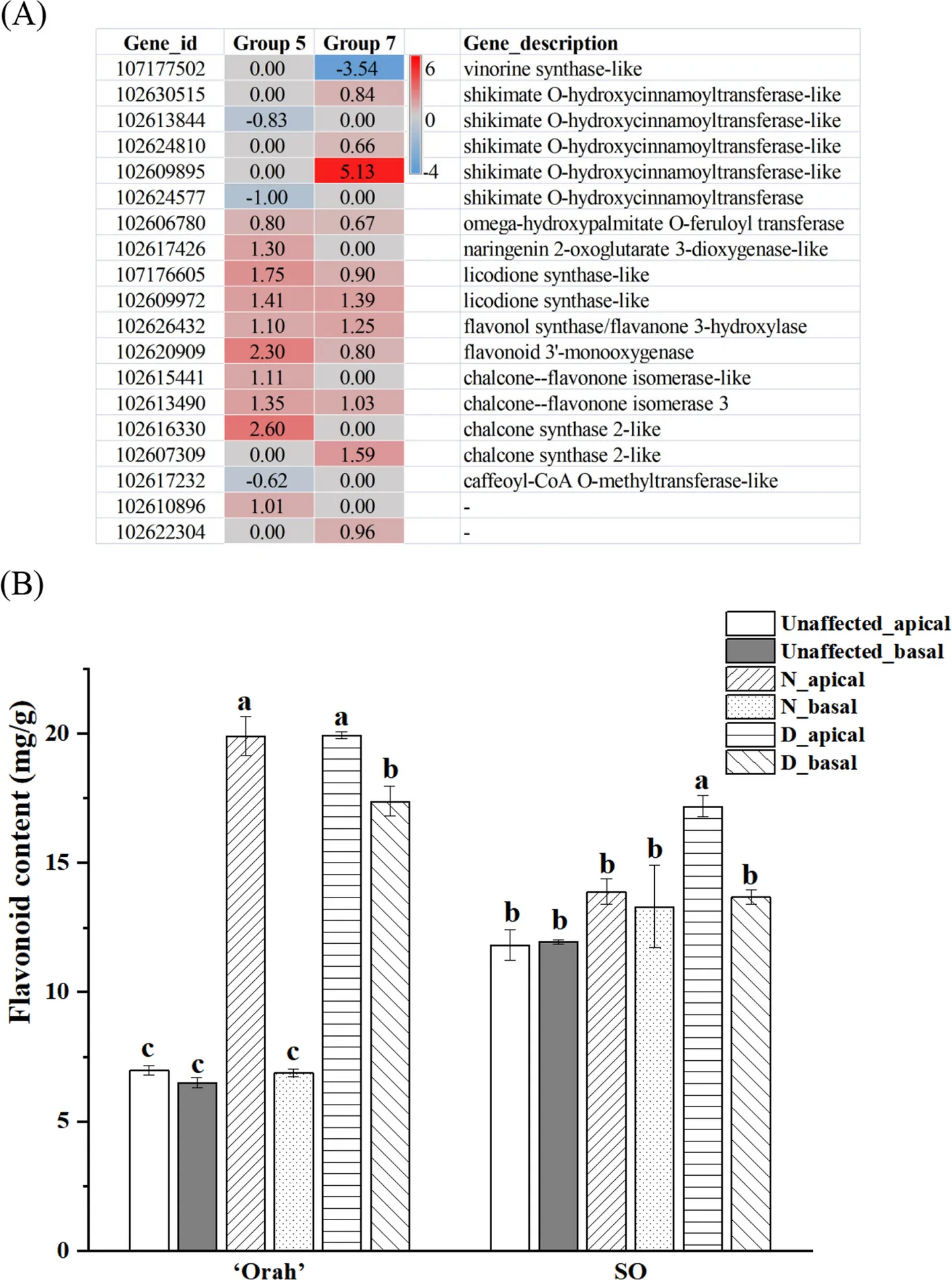
Log_2_fold change cluster diagram of DEGs in the flavonoid biosynthesis pathway and histogram of flavonoid content in leaves. **A** Log_2_fold change cluster diagram of the DEGs in the flavonoid biosynthesis pathway. **B** flavonoid content of sweet orange (SO) and ‘Orah’. D Huanglongbing-affected. N Zn/Mn-deficiency-affected. Apical younger mature (upper) leaves. Basal older mature (lower) leaves. Group 5 SO_D_apical *vs.* SO_D_basal. Group 7 ‘Orah’_D_apical *vs.* ‘Orah’_D_basal. All data are presented as means ± standard deviation of the mean (SD), error bars represent the SD. Bars with different letters indicate significant differences at the *p* < 0.05 level

KEGG analysis revealed flavonoid biosynthesis (cit00941) was specifically enriched in HLB-affected apical leaves than basal leaves in SO (13 DEGs in Groups 5) and ‘Orah’ (12 DEGs in Group 7), with 10 and 11 upregulated genes respectively, including key enzymes like chalcone synthases and flavanone 3-hydroxylases (Fig. 8A). Of the DEGs, genes encoding licodione synthase-like, flavonol synthase/flavanone 3-hydroxylases, chalcone–flavonone isomerases, chalcone synthases were noteworthy. To validate these transcriptomic findings at the metabolic level, we measured flavonoid content in corresponding leaf samples. Physiological measurements showed that flavonoid levels were consistently higher in apical leaves than in basal leaves under HLB infection in both cultivars. While nutrient-deficient ‘Orah’ also showed this apical–basal difference, it was not observed in nutrient-deficient sweet orange. No significant differences were detected between apical and basal leaves in unaffected controls of either cultivar (Fig. 8B).

#### Activation of glutathione transferase family genes in fully mature leaves of both cultivars

**Fig. 9:**
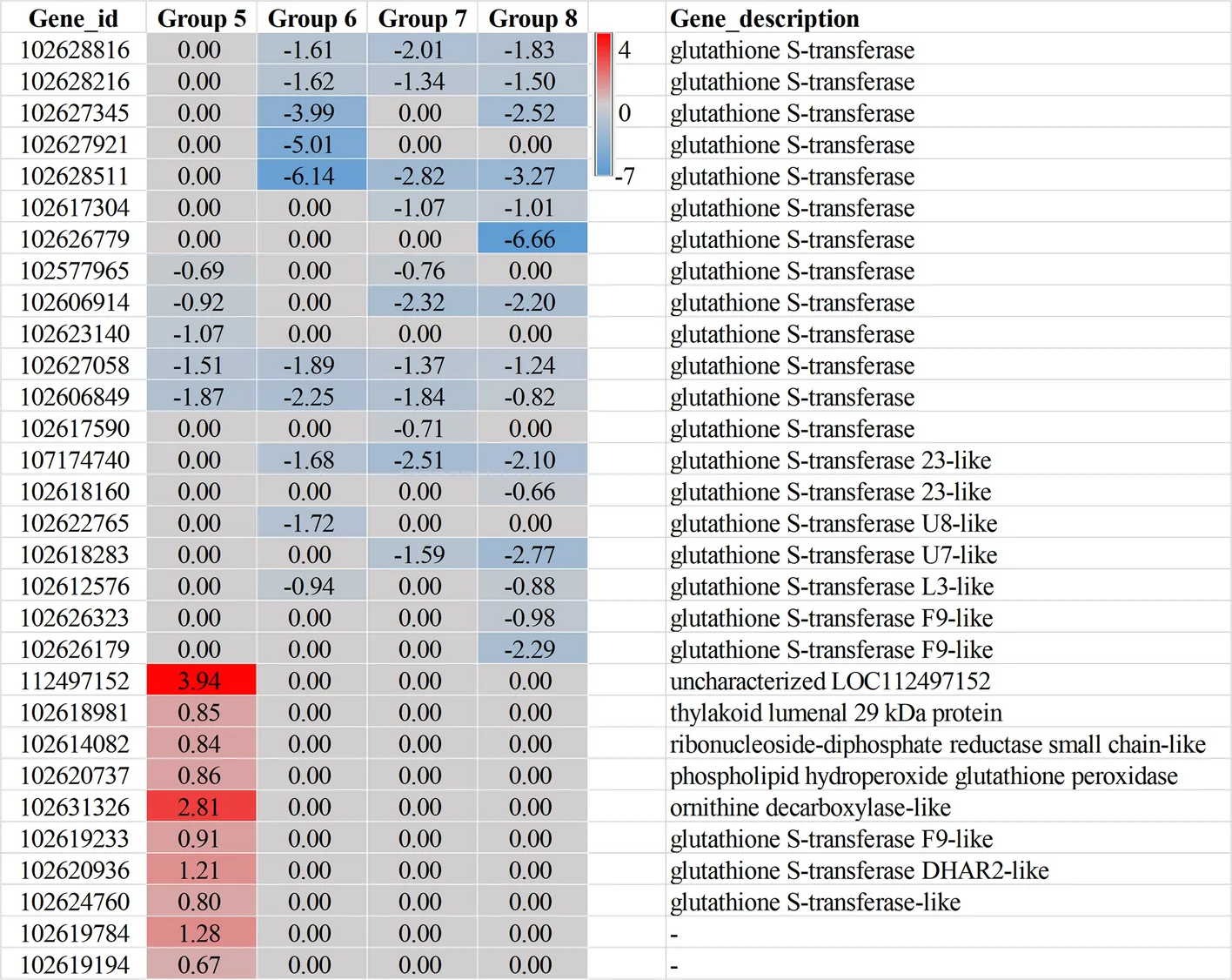
Log_2_fold change cluster diagram of DEGs in the glutathione metabolism pathway in upper leaves compared to lower leaves. Detailed information regarding the comparison groups can be found in Table 1

Based on KEGG analysis results, alterations in the transcriptome of glutathione S-transferase (GST) across different leaf ages in response to CLas infection or Zn/Mn deficiency induction were screened (Fig. 9). Notably, none of the 20 down-regulated GST genes belonging to Group 6, 7, or 8 were upregulated in Group 5. Conversely, the remaining 10 GST-related DEGs (comprising three GST genes and three uncharacterized genes) were exclusively upregulated in Group 5 and did not exhibit differential expression in the other three groups.

#### Differential regulation of amino acid metabolism and terpenoid-quinone biosynthesis in leaves of different ages in sweet orange, but not in ‘Orah’

**Fig. 10:**
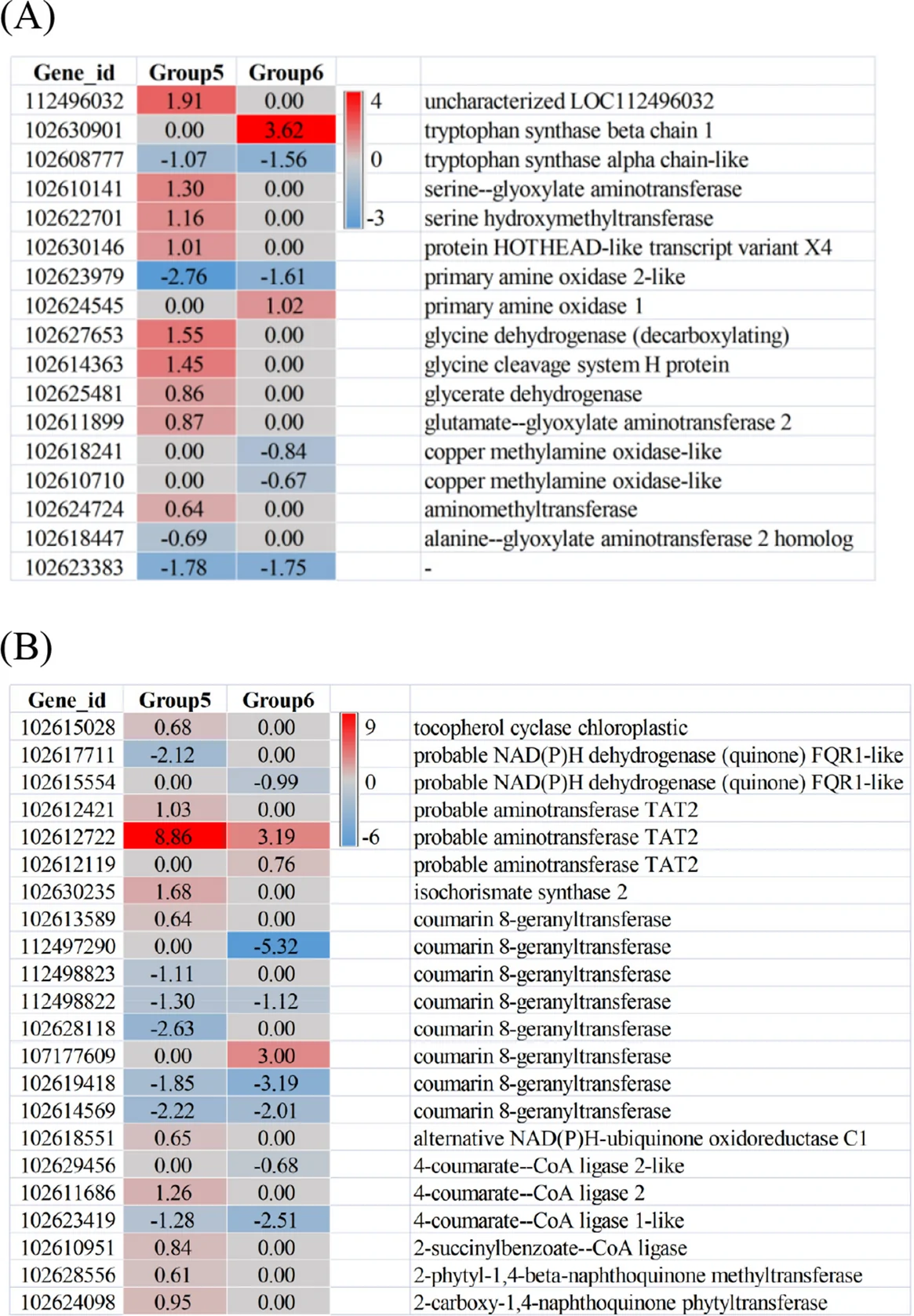
Log_2_fold change cluster diagram of DEGs in glycine, serine, and threonine metabolism pathway and ubiquinone and other terpenoid-quinone biosynthesis pathway. **A** represents the Log_2_fold change cluster diagram of DEGs in the glycine, serine, and threonine metabolism pathway. **B** represents the Log_2_fold change cluster diagram of DEGs in the ubiquinone and other terpenoid-quinone biosynthesis pathway. Group 5 corresponds to SO_D_apical *vs.* SO_D_basal. Group 6 corresponds to SO_N_apical *vs.* SO_N_basal

KEGG analysis showed significant enrichment of "glycine, serine, and threonine metabolism (cit00260)" (Fig. 10A) and "Ubiquinone and other terpenoid-quinone biosynthesis (cit00130)" (Fig. 10B) in Groups 5–6, but not in Group 7 or Group 8. Specifically, in Group 5, 17 and 13 DEGs were classified into cit00130 and cit00260, respectively. For Group 6, the corresponding numbers were 10 and 7 DEGs. Although Groups 7–8 showed some DEGs for cit00130 (10 and 9), these pathways weren't significantly enriched. Subsequent analysis focused on 22 cit00130-related DEGs (Fig. 10B) and 17 cit00260-related DEGs (Fig. 10A) in Groups 5 and 6, revealing upregulation of TAT2 aminotransferases and downregulation of coumarin 8-geranyltransferases.

### Summary of key differentiating features between HLB and Zn/Mn deficiency

Our integrated analysis delineates a set of defining features that robustly distinguish HLB-induced chlorosis from Zn/Mn deficiency. At the molecular level, HLB infection triggered a profound and specific suppression of photosynthetic machinery and a marked accumulation of leaf starch, responses that were significantly more pronounced than those observed under nutrient deficiency. Most distinctively, HLB, but not nutrient deficiency, consistently activated biotic stress pathways, including phenylpropanoid/flavonoid biosynthesis and plant-pathogen interactions. Physiologically, only HLB led to significant reductions in leaf biomass and severe deterioration of fruit quality. In contrast, primary Zn/Mn deficiency primarily manifested as reduced leaf concentrations of the corresponding micronutrients without triggering these systemic biotic defense responses or causing substantial impacts on biomass and fruit quality. These contrasting signatures provide a multi-dimensional basis for accurate differentiation in the field.

## Discussion

This study was explicitly designed to address a specific agronomic challenge: differentiating HLB from Zn/Mn deficiency based on their nearly identical chlorosis symptoms. Therefore, our transcriptomic analyses were structured as a direct, head-to-head comparison between these two confounding conditions. While enhanced photosynthesis typically correlates with increased biomass and crop yields [20, 21], our study reveals that, despite the similarity in chlorotic symptoms, CLas infection induces more severe and specific suppression of photosynthesis and distinct transcriptomic alterations compared to Zn/Mn deficiencies. The unreliable correlation between leaf and soil nutrient status in the field [3] necessitates the use of physiological markers like chlorophyll fluorescence for timely and accurate differentiation of nutrient deficiencies from HLB. Besides, the genes commonly induced in by HLB, as shown in Fig. 6, will undergo further screening to facilitate the distinction between leaf samples affected by these two disorders. Transcriptome analysis demonstrates CLas-induced downregulation of key photosynthetic genes (chlorophyll a-b binding proteins, PSI/PSII subunits) critical for light energy conversion [8], confirming photosynthetic inhibition despite tissue-specific variations reported previously [8, 9, 22, 23]. Notably, recent studies using sun-induced chlorophyll fluorescence (SIF) further support this disruption [24]. The observed disconnect between leaf yellowing severity and both photosynthetic capacity and biomass production suggests biotic stress (CLas infection) causes more severe physiological disruption than comparable abiotic stress (Zn/Mn deficiency). Importantly, this profound and specific suppression of photosynthetic, captured by SIF signatures, was uniquely severe and consistent in CLas infection, forming a clear contrast to the milder and more variable impact observed under Zn/Mn deficiency. This divergence underpins the utility of remote sensing for early, differential diagnosis [24].

Consistent with the photosynthetic level, the carbon metabolism level of HLB-affected ZDL leaves was elevated compared to that of HLB-affected BM leaves. This observation was supported by the upregulation of genes encoding alcohol dehydrogenase and sedoheptulose-1,7-bisphosphatase (SBPase). Alcohol dehydrogenase has been induced under stresses such as drought, low temperature, and pathogen invasion [25]. Previous studies have demonstrated that overexpression of the gene encoding SBPase successfully altered the photosynthetic rate in *Arabidopsis thaliana* [26]. The upregulation of carbon assimilation genes (e.g., SBPase) in HLB-affected leaves suggests that citrus may activate a compensatory metabolic response to mitigate infection-induced photosynthetic suppression, a response pattern not observed, or observed to a much lesser extent, in Zn/Mn-deficient leaves. This highlights a divergent metabolic strategy under biotic versus abiotic stress.

Disruptions in carbon metabolism resulting from alterations in photosynthesis directly impede the carbohydrate balance in plants [27], thereby influencing plant growth and biomass accumulation [28]. Citrus plants infected with CLas exhibit excessive starch accumulation in their leaves, which disrupts the chloroplast thylakoid structure and results in a substantial decline in photosynthesis [29, 30]. Crucially, the classic HLB hallmark of excessive starch accumulation was markedly more pronounced than in Zn/Mn-deficient leaves, reaffirming this metabolic dysfunction as a core, persistent feature of the biotic stress when directly contrasted with abiotic deficiency. Correspondingly, genes encoding the large subunit of glucose-1-phosphate adenylyltransferase, granule-bound starch synthase, and alpha-glucan, which are associated with starch synthesis [31–33], were up-regulated in response to CLas infection. Despite the inhibition of photosynthesis, starch accumulation and sucrose elevation were observed in HLB-affected leaves. Cai et al. and Freitas et al. also reported that the sucrose content in HLB-affected leaves was significantly higher than that in normal leaves [34, 35]. We propose that the alteration in sucrose content is directly linked to the photosynthesis synthesis process, while starch accumulation may be related to the deceleration of starch degradation. This metabolic signature serves as a reliable diagnostic marker to differentiate HLB-induced chlorosis from nutrient deficiency symptoms. Polyploid Citrus changshan-huyou similarly exhibits metabolic shifts in flavonoids and amino acids [36], suggesting conserved stress adaptation mechanisms between genomic doubling and pathogen response, albeit with tissue-specific expression patterns. While leaf starch accumulation is a well-established indicator of HLB [37, 38], our study provides critical evidence for its diagnostic specificity in a comparative context. Notably, our finding—that Zn/Mn deficiency in citrus does not trigger leaf starch accumulation—contrasts with reports in annual crops like rice, where Zn deficiency can induce starch deposition in vegetative tissues as a potential metabolic adaptation [39]. However, this response is not universal, as Zn status does not always correlate directly with grain starch content [40], though foliar Zn application can enhance starch accumulation under deficiency [41]. This divergence likely reflects species-specific differences in carbohydrate partitioning and Zn physiology between woody perennials and herbaceous plants. Our results therefore reinforce that leaf starch accumulation is a stress response highly specific to HLB in citrus, rather than a general marker of nutrient deficiency.

HLB-affected citrus plants exhibit significantly reduced Zn concentrations [42], a finding confirmed in our study. Exogenous application of Zn^2+^ enhances the antibacterial capacity of plants and improves Zn absorption by citrus leaves [43, 44]. However, Zhang et al. observed that despite an increase in Zn content in leaves following application, HLB symptoms were not alleviated [45]. In fact, low Zn concentrations can promote the growth of CLas, and only when Zn concentrations in phloem tissue reach a threshold level do they contribute to the elimination of HLB symptoms. CLas-ZnuA2, the second binding protein of the Znu system in CLas, exhibits low binding affinity for Mn^2+^ and Zn^2+^, suggesting that the presence of CLas is detrimental to the accumulation of these ions [46]. Notably, Mn^2+^ and Zn^2+^ concentrations were significantly lower in nutrient-deficient leaves compared to unaffected leaves, contrasting with iron deficiency which is readily corrected by fertilization—underscoring the greater challenge of managing biotic versus abiotic stress. Our data caution that foliar Zn application alone is unlikely to reverse HLB symptoms, as the pathogen disrupts Zn homeostasis. Nutrient management should focus on maintaining overall tree vigor to support defense responses, rather than expecting a direct therapeutic effect on HLB.

Plants defend themselves against pathogen attacks by inducing appropriate defense responses [47]. The identification of conserved NAC domain-containing proteins among DEGs mirrors findings in cold-stressed citrus, where PtrNAC2 negatively regulates tolerance [48], suggesting NACs may universally mediate stress response trade-offs in citrus. The secondary metabolite phenylpropanoid, which is ubiquitous in plants, plays a pivotal role in the defense mechanism against pathogens [49]. In HLB-affected citrus, we observed differential expression of key phenylpropanoid genes (caffeic acid 3-O-methyltransferase, shikimate O-hydroxycinnamoyltransferase, and peroxidases) between Zn/Mn-deficient and CLas-infected leaves. These enzymes enhance resistance by regulating lignin synthesis [50–52], with higher lignin levels correlating with reduced disease incidence and improved stress tolerance [53, 54]. Bioinformatics analysis has revealed the presence of approximately 80 peroxidase genes (13 class I and 67 class III) in *Citrus sinensis*. The responsiveness of these key defense components during CLas infection can be influenced by plant genotype and/or environmental factors, resulting in varied expression profiles among specific genes [55]. Consequently, the phenylpropanoid metabolism-related plant defense mechanisms against biotic and abiotic stresses are synergistic and complex. GSTs are key multifunctional enzymes in plant stress adaptation, involved in detoxification and redox regulation [56]. We found the GSTs are age-dependent and variety-specific, not consistently linked to infection status alone (mostly activated in mature basal leaves). These findings highlight the dual, context-dependent roles of GSTs and support their use as physiological markers to differentiate abiotic from biotic stress, offering potential targets for breeding resilient citrus varieties. Critically, our direct comparison reveals that the sustained and coordinated upregulation of this defense pathway is a distinctive response to CLas infection, setting it apart from the more limited or absent induction in Zn/Mn-deficient leaves. The sustained upregulation of phenylpropanoid biosynthesis genes in HLB-affected leaves identifies key enzymatic steps as potential biomarkers for selecting HLB-tolerant rootstocks or scions in breeding programs.

Flavonoid is a kind of plant derived compound with antibacterial activity and strong antioxidant ability, which is related to the resistance of citrus to CLas infection [57, 58]. In HLB-affected shoots, the flavonoid biosynthesis pathway was significantly enriched, with upregulated genes (chalcone synthase, flavonoid 3'-monooxygenase, and flavonol synthase) in ZDL leaves indicating that flavonoid biosynthesis in ZDL leaves was stronger than that in BM leaves. Our biochemical assays showed that flavonoid biosynthesis is not an exclusive response to HLB, as it was also upregulated in Zn/Mn-deficient ‘Orah’ leaves at the metabolic level. However, the transcriptional induction of this pathway was more consistently and strongly enriched in HLB-affected leaves across both cultivars and leaf ages (Group 5 and 7). As flavonoids also mediate abiotic stress responses (e.g., UV protection via their sunscreen properties) [59], their accumulation suggests younger leaves mount stronger defenses against both biotic (CLas) and abiotic (nutrient) stresses than mature leaves. Furthermore, our study reveals significant activation of the plant-pathogen interaction pathway in CLas-infected BM leaves compared to Zn/Mn-deficient leaves, with varietal differences in response intensity. This biotic stress response integrates both PAMP-triggered immunity (PTI) and Effector-triggered immunity (ETI) immune mechanisms, demonstrating stronger and more coordinated defense activation than observed under abiotic stress conditions. The specific activation of the plant-pathogen interaction pathway in HLB provides a molecular basis for monitoring defense competency in orchards. Coupled with flavonoid levels, these signatures could be used to assess the efficacy of therapeutic treatments (e.g., antimicrobials, phytoalexin) aimed at boosting tree immunity.

In summary**,** acknowledging that greenhouse studies with controlled inoculations and nutrient regimes represent the gold standard for unequivocally separating these effects, our field-based study provides a foundational transcriptomic dataset that should be validated under such controlled conditions. Nevertheless, this work successfully delineates the molecular divergence between HLB and Zn/Mn deficiency and translates these findings into actionable insights for citrus management. Our comparative analysis reveals that while both conditions share common features such as chlorosis and alterations in nutrient-responsive genes—likely a consequence of HLB-induced nutrient dysregulation—the key distinction lies in the sustained and specific activation of biotic stress pathways (e.g., plant-pathogen interaction, phenylpropanoid/flavonoid biosynthesis) in HLB-infected leaves, which are minimal or absent in leaves with primary nutrient deficiency even when both exhibit nutrient imbalance. Based on this mechanistic insight, we propose a complementary diagnostic approach: SIF, targeted starch testing, and molecular signatures of biotic defense offer confirmatory evidence in difficult cases. Furthermore, the identified defense-related genes offer novel targets for breeding resilient varieties and developing immune-boosting therapies. By moving from descriptive biology to solution-oriented recommendations, this work provides a concrete framework for improving HLB mitigation in the field.

## Materials and methods

### Plant materials

**Fig. 11:**
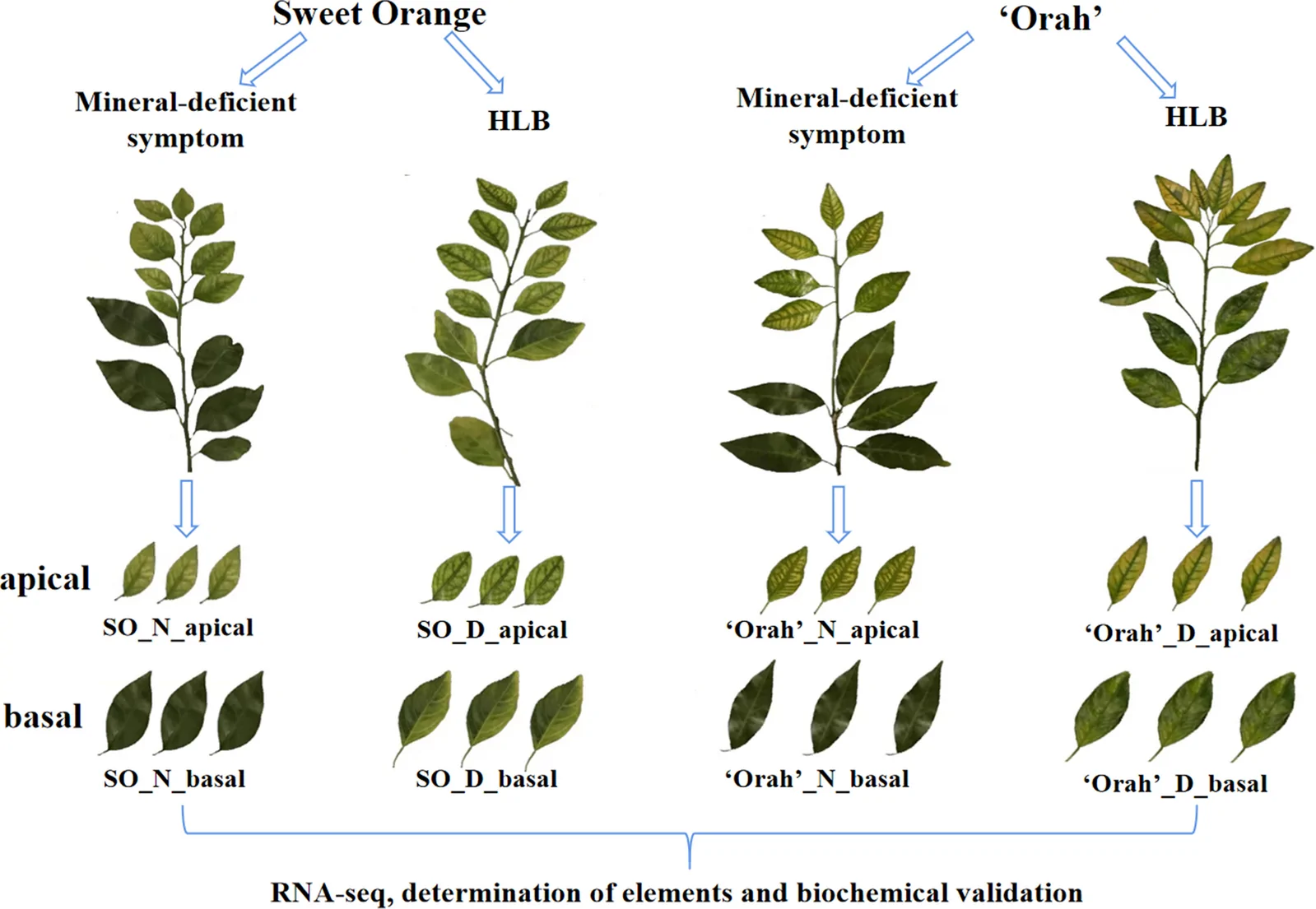
Schematic of sampling design for HLB-affected and nutrient-deficient citrus leaves. ‘Orah’ *Citrus reticulata* Blanco ‘Orah’. SO sweet orange (*Citrus sinensis* (L.) Osbeck). D CLas-infected symptomatic shoots. N abiotic nutrient-deficient shoots. Apical younger mature upper leaves. Basal older mature lower leaves

Plant samples were collected from fruit-bearing trees in two commercial citrus orchards in southern China during the typical fruit-bearing season when deficiency symptoms were fully expressed: one located at 114°28'E, 23°29'N, with 10-year-old sweet orange trees, and the other at 113°29'E, 23°38'N, with 6-year-old ‘Orah’ trees. From each orchard, ten HLB-symptomatic trees (showing BM or ZDL chlorosis) and ten presumed nutrient-deficient trees (exhibiting nutrient deficiency symptoms but testing CLas-negative via PCR) were selected. Five shoots with uniform chlorosis were sampled per tree. Both younger mature leaves (apical) and older mature leaves (basal) from the same shoot were collected. Samples were systematically categorized based on citrus variety, stress type (biotic/abiotic), and leaf position (apical/basal). For sweet orange, CLas-infected shoots were labeled as SO_D_apical (upper leaves) and SO_D_basal (lower leaves), while nutrient-deficient shoots were designated SO_N_apical and SO_N_basal. Similarly, for ‘Orah’ mandarin, samples were identified as ‘Orah’_D_apical, ‘Orah’_D_basal, ‘Orah’_N_apical, and ‘Orah’_N_basal (Fig. 11). Each group included three biological replicates. All samples were flash-frozen in liquid nitrogen and stored at − 80 °C for downstream RNA extraction, CLas detection, and physiological assays.

### Determination of starch, sucrose and chlorophyll contents

Fresh leaf tissue (100 mg) was homogenized for sucrose analysis using a Plant Sucrose Content Detection Kit (Solarbio Science & Technology, Beijing, China) following Xie et al. [60]. Starch content was measured from 30 mg of fresh tissue using a Plant Starch Content Detection Kit (Solarbio), adhering to the manufacturer’s protocol. Chlorophyll extraction was performed by grinding 100 mg of fresh leaves in 80% acetone, by measuring absorbance at specific wavelengths with UV–Vis Spectrophotometry and applying established Lichtenthaler’s formula [61].

### DNA extraction and PCR detection of CLas

Genomic DNA was extracted from ~ 100 mg of leaf tissue using the E.Z.N.A.® Plant DNA Kit (Omega Bio-Tek, Norcross, GA, USA) and quantified with a Qubit® 2.0 Fluorometer (Thermo Fisher Scientific Inc., Waltham, MA, USA). CLas detection was performed via qPCR targeting the 16S rDNA region [62]. Reactions contained 100 ng DNA template and were run on a CFX Connect Real-Time System (Bio-Rad, USA). Samples with quantification cycle (Cq) values < 34 were classified as CLas-positive, based on positive (Cq < 30) and negative (Cq > 38) controls.

### RNA preparing, sequencing and transcriptome analysis

A direct comparative transcriptomic design was employed between HLB-affected and Zn/Mn-deficient leaves to specifically identify genes that are differentially expressed between these two visually similar conditions—the core challenge for field diagnosis. Total RNA was extracted from liquid-nitrogen-ground tissues of eight treatment groups × three biological replicates = 24 total samples. Each replicate represented an independent tree, using the E.Z.N.A.® Plant RNA Kit (Omega Bio-tek, Inc., Norcross, GA, USA). RNA integrity and purity were verified using the RNA Nano 6000 Assay Kit on a Bioanalyzer 2100 system(Agilent Technologies, CA, USA), with all samples meeting RNA-seq quality criteria (Table S1).

For library construction, polyadenylated mRNA was enriched from 1 μg total RNA per sample using Oligo(dT) magnetic beads. Following fragmentation in divalent cation buffer (94 °C, 5 min), cDNA synthesis was performed using M-MuLV Reverse Transcriptase (RNase H-) for first-strand synthesis and DNA Polymerase I/RNase H for second-strand synthesis. After end repair and 3' adenylation, Illumina adapters were ligated to 370–420 bp fragments selected through AMPure XP bead purification (Beckman Coulter, Beverly, USA). PCR amplification was conducted using Phusion High-Fidelity DNA polymerase with index primers, followed by quality verification on the Bioanalyzer 2100. Libraries were clustered on a cBot system (TruSeq PE Cluster Kit v3) and sequenced on an Illumina Novaseq platform (150 bp paired-end).

Raw sequencing data were preprocessed by: 1) Removing adapter-contaminated reads using Cutadapt; 2) Filtering low-quality reads (Qscore < 20 in > 50% bases) and poly-N sequences (> 10% N content) through in-house Perl scripts; 3) Calculating Q20/Q30 scores and GC content. Clean reads were aligned to the *Citrus sinensis* reference genome (GCA_00031741.5) using Hisat2 v2.0.5 with default parameters. Transcript assembly and quantification were performed using StringTie v1.3.3b in reference-based mode, with gene-level counts obtained through FeatureCounts v1.5.0-p3.

Differential expression analysis was conducted in DESeq2 (v1.20.0) using normalized FPKM values. Two analytical frameworks were applied: 1) For HLB vs nutrient deficiency comparisons (Groups 1–4), DEGs were defined as |log_2_FC|≥ 1 with FDR-adjusted p < 0.05 (Benjamini–Hochberg method); 2) For leaf age comparisons (Groups 5–8), a moderated threshold of |log_2_FC|≥ 0.58 (equivalent to 1.5-fold change) with identical FDR control was employed. Significant DEGs underwent functional enrichment analysis using clusterProfiler with GO and KEGG databases, incorporating gene length bias correction.

### Leaf mineral content measurement

Leaf mineral content (Ca, Fe, K, Mg, Mn, Mo, P, S, Zn) was quantified in five 'Orah' mandarin leaf types: CLas-infected upper (D_apical) and lower (D_basal) leaves, nutrient-deficient upper (N_apical) and lower (N_basal) leaves, and unaffected controls (n = 3 biological replicates per group). Approximately 0.3 g of lyophilized leaf tissue per sample underwent microwave-assisted digestion (Preekem Scientific Instruments Co., Ltd., Shanghai, China) with 5 mL concentrated HNO_3_ followed by dilution to 10 mL with 1% HNO_3_. Elemental quantification was performed using inductively coupled plasma mass spectrometry (iCAP TQ ICP-MS, ThermoFisher, Waltham, USA) in collision cell technology mode (He/O_2_ gas mixture), following established protocols [63, 64]. Instrument calibration included NIST SRM certified reference material and blank controls.

### Phenotypic characterization of the leaves and fruits

Three phenotypically uniform leaf cohorts (D, U, H; n = 3 biological replicates) and corresponding fruits were analyzed. Leaf dimensions (length × width) and fresh weight were measured using digital calipers (Lulin Tools Co., Ltd., Yantai, Shandong, CN) and an analytical balance (Zhengfeng Electronic Technology Co., Ltd. Suzhou, CN), respectively. Fruit parameters were assessed through: 1) Juice yield: Mechanical extraction (Joyoung laboratory juicer) followed by triple-layer cheesecloth filtration.

2) Total soluble solids: Digital refractometer (Shenzhen Liushu Technology Co., Ltd., Shenzhen, CN). 3) Titratable acidity: pH meter (Langeke Technology Co., Ltd., Beijing, CN) via NaOH titration to pH 8.2 endpoint. 4) Ascorbic acid: Modified 2,6-dichlorophenolindophenol spectrophotometric method [65]. All measurements included three technical replicates.

### Transcriptomic validation

Twenty functionally diverse DEGs were selected for RT-qPCR validation using RNA-seq-derived templates. Gene-specific primers (Table S2) were designed with Primer-BLAST (NCBI) following MIQE guidelines. Reverse transcription employed the reverse transcription employed the TransScript® with gDNA eraser (TransGen Biotech, Beijing, CN). Quantitative PCR was performed on the Applied Biosystem (Thermo Fisher Scientific, WLM, USA) using SYBR Green chemistry, with GAPDH as the reference gene (ΔΔCq method). Amplification efficiency (90–110%) and melt curve analysis confirmed primer specificity. The Log_2_fold change of RT-qPCR for each gene was compared with the corresponding RNA-seq data from the same sample (Table S3).

### Data analysis

All Data of the above experiments were presented as means ± SD. Statistical significance (*p* < 0.05) was determined using one-way ANOVA with Duncan’s multiple range test in SPSS v13.0, with differences denoted by lowercase letters in figures.

## Supplementary Information

Supplementary Material 1.

Supplementary Material 2.

## Data Availability

The datasets generated and/or analyzed during the current study are available in the National Genomics Data Center [(https:/ngdc.cncb.ac.cn)] with the primary accession code PRJCA040964. All data generated or analyzed during this study are included in this published article and its supplementary information files. All materials are available in the lab.
