# Supplementary material for: Integrated physiological and transcriptomic analysis reveals molecular distinctions between Huanglongbing- and Zn/Mn deficiency-induced chlorosis in citrus
Source: BMC Plant Biol. 2026 Aug 8;26:1890. doi: 10.1186/s12870-026-09447-0 (PMC13737685; doi:10.1186/s12870-026-09447-0)
Supplement: Supplementary file 2 [file 12870_2026_9447_MOESM2_ESM.docx]

Table S1 Quality indicators of total RNA for RNA-seq

| **Sample** | **Concentration**  **(ng/ul)** | **Total**  **(ul)** | **Quantum**  **(ug)** | **Integrity**  **Value** | **Test Conclusions** |
| --- | --- | --- | --- | --- | --- |
| SO_D1_up | 60 | 35 | 2.10 | 8.90 | A |
| SO_D1_down | 87 | 35 | 3.05 | 9.20 | A |
| SO_D2_up | 22 | 35 | 0.77 | 8.80 | A |
| SO_D2_down | 28 | 35 | 0.98 | 9.80 | A |
| SO_D3_up | 70 | 35 | 2.45 | 9.10 | A |
| SO_D3_down | 20 | 35 | 0.70 | 8.80 | A |
| SO_U1_up | 29 | 35 | 1.02 | 8.30 | A |
| SO_U1_down | 83 | 35 | 2.91 | 8.40 | A |
| SO_U2_up | 318 | 35 | 11.13 | 7.30 | A |
| SO_U2_down | 56 | 35 | 1.96 | 8.00 | A |
| SO_U3_up | 56 | 35 | 1.96 | 8.40 | A |
| SO_U3_down | 488 | 35 | 17.08 | 7.10 | A |
| ‘Orah’_D1_up | 58 | 35 | 2.03 | 8.40 | A |
| ‘Orah’_D1_down | 53 | 35 | 1.86 | 8.30 | A |
| ‘Orah’_D2_up | 34 | 35 | 1.19 | 9.10 | A |
| ‘Orah’_D2_down | 58 | 35 | 2.03 | 8.50 | A |
| ‘Orah’_D3_up | 76 | 35 | 2.66 | 8.90 | A |
| ‘Orah’_D3_down | 58 | 35 | 2.03 | 8.20 | A |
| ‘Orah’_U1_up | 41 | 35 | 1.44 | 8.20 | A |
| ‘Orah’_U1_down | 82 | 35 | 2.87 | 8.30 | A |
| ‘Orah’_U2_up | 27 | 35 | 0.95 | 8.40 | A |
| ‘Orah’_U2_down | 64 | 35 | 2.24 | 8.50 | A |
| ‘Orah’_U3_up | 64 | 35 | 2.24 | 8.10 | A |
| ‘Orah’_U3_down | 39 | 35 | 1.37 | 8.20 | A |

Note: SO: Sweet orange (*Citrus sinensis* (L.) Osbeck); D: Huanglongbing-affected; U: Mineral nutrient-deficiency-affected; Up: Younger mature (upper) leaves; Down: Older mature (lower) leaves. E.g., 'SO_D_up' denotes the younger mature leaves of sweet orange shoots infected with CLas.

Table S2 Primers for the 20 DEGs and GAPDH

| **Gene_id** | **Primer** |
| --- | --- |
| GAPDH | Forward primer TGCTCCCATGTTTGTTGTGG Reverse primer AAGAGGAGCTAGGCAGTTGG |
| 102625194 | Forward primer CTGTCTGGCCCATACCACAG  Reverse primer ACCTTAACCTACACCTTCAGCC |
| 102616003 | Forward primer AGGGGAGACGGTAGTCAACA  Reverse primer TTGACAAGTTTCGTGCGCTG |
| 102629211 | Forward primer AAGCGATTTGTCCTGTTTGGC  Reverse primer TTGCATGGGAAGCGTGGTTT |
| 102616774 | Forward primer GCCGTTTTGTGGCTTTCACT  Reverse primer TGCCACAAGATGCACTAGCA |
| 102626207 | Forward primer GTTTCGCACATTGTTGGCCT  Reverse primer CACCAGTGCACCAATCAACA |
| 102623486 | Forward primer TCGATTTAGCCCAGCATCCG  Reverse primer TATGTGGACGCAGGTATGGC |
| 102610533 | Forward primer TGTCTCGGGGTCTGCTGATA  Reverse primer AATCGACAAACAGGTACGGC |
| 102622879 | Forward primer TCAGCTGAGGTTCAGTGACTAC  Reverse primer GCTTTTTGACTCGGTGAAGGT |
| 102623755 | Forward primer AGAATCCTGGGGCCTGTACT  Reverse primer ATTTGGCACAGGGATGAACT |
| 102617251 | Forward primer GGGGCATGCATCTTCGACTA  Reverse primer CTTCGCTTTGCATCTCAGGG |
| 102622791 | Forward primer GAGATGGCCCATTCCACTGT  Reverse primer TGTTCAGGCACACGATTTCT |
| 102628301 | Forward primer GTCCAAAGCCTAATAAAGCACCA  Reverse primer AGCAACTCCTCTGATGTGCG |
| 102607393 | Forward primer TGAGCATCGAGCTTGCTCTT  Reverse primer GATCTTTGGAGCCCTCGGTT |
| 102617875 | Forward primer TCGTGGAAGGTTGTCTGCAA  Reverse primer CCTACGCTCTCACACTAACCC |
| 102628588 | Forward primer CACCTTGGATCCCTCGCTAC  Reverse primer CACTCTTTATTGTTGTAGGCCGT |
| 102621104 | Forward primer GGCAATGGCAGAACTGCTTC  Reverse primer GCCTACCACTTGCTCCAACT |
| 102625945 | Forward primer AAGTCCAAGCTTAGCCCGAG  Reverse primer GGATGGTGAAAAAGTCCGGC |
| 102612722 | Forward primer ATGCTTGATGTTTGCAGGGC  Reverse primer CCTCCCAAGGCCATCTTCAA |
| 102609895 | Forward primer TTGGATCAACAGGCGAGAGG  Reverse primer GGGGCCTTGTAGCAACTCAT |
| 102628816 | Forward primer GAAGAGACGTGGCCACAGAA  Reverse primer CCAGAGTTGTGAGCGAACCT |

Table S3 The detail information of 20 DEGs

| **Gene_id** | **log_2_Fold change** | | **Gene_description** |
| --- | --- | --- | --- |
|  | **qPCR** | **RNA-seq** |  |
| 102625194 | -1.63 ± 0.20 | -1.43 | Chlorophyll a-b binding protein 13; chloroplastic |
| 102616003 | 1.86 ± 0.27 | 1.56 | Sucrose synthase; transcript variant X1 |
| 102629211 | 2.47 ± 0.31 | 3.13 | Glucose-1-phosphate adenylyltransferase large subunit 1-like |
| 102616774 | 2.94 ± 0.48 | 2.08 | Isoamylase 1; chloroplastic |
| 102626207 | 2.22 ± 0.14 | 1.37 | Isoamylase 2; chloroplastic |
| 102623486 | -0.74 ± 0.17 | -1.22 | Cytochrome P450 90B transcript variant X1 |
| 102610533 | 1.27 ± 0.20 | -1.53 | Chlorophyll a-b binding protein 151; chloroplastic |
| 102622879 | -1.57 ± 0.26 | -3.21 | Cytochrome P450 CYP749A22-like transcript variant X1 |
| 102623755 | -1.08 ± 0.25 | -2.3 | Cytochrome P450 CYP749A22-like |
| 102617251 | 2.29 ± 0.23 | 4.02 | Peroxidase 3 |
| 102622791 | 1.04 ± 0.14 | -1.33 | Cytochrome P450 71A1-like |
| 102628301 | 3.45 ± 0.55 | 3.6 | Heat shock protein 83 |
| 102607393 | 4.20 ± 0.07 | 4.1 | Beta-glucosidase 46-like |
| 102617875 | 4.39 ± 0.41 | 3.91 | Glucan endo-3-beta-glucosidase-like |
| 102628588 | 5.99 ± 0.06 | 6.46 | Beta-xylosidase/alpha-L-arabinofuranosidase 1 |
| 102621104 | -0.81 ± 0.14 | 4.99 | Flavonoid 3'-monooxygenase-like |
| 102625945 | 2.23 ± 0.13 | 4.19 | Serine carboxypeptidase-like 17 |
| 102612722 | 3.46 ± 0.31 | 4.29 | Probable aminotransferase TAT2 |
| 102609895 | 1.47 ± 0.27 | 1.59 | Chalcone synthase 2-like |
| 102628816 | -1.78 ± 0.15 | -2.01 | Glutathione S-transferase |

Table S4 Sequence data and quality of RNA seq

| **Sample** | **Raw_reads** | **Clean_reads** | **Clean_bases** | **Error_rate** | **Q20** | **Q30** |
| --- | --- | --- | --- | --- | --- | --- |
| SO_D1_up | 45429572 | 43213526 | 6.48 G | 0.03 | 97.36 | 92.37 |
| SO_D1_down | 46823678 | 45019976 | 6.75 G | 0.03 | 97.12 | 91.85 |
| SO_D2_up | 43318676 | 40838436 | 6.13 G | 0.03 | 97.40 | 92.57 |
| SO_D2_down | 47266658 | 44939234 | 6.74 G | 0.03 | 97.14 | 91.90 |
| SO_D3_up | 42470264 | 40327634 | 6.05 G | 0.03 | 97.12 | 91.85 |
| SO_D3_down | 41493360 | 39517972 | 5.93 G | 0.03 | 97.16 | 91.92 |
| SO_U1_up | 42623572 | 40716514 | 6.11 G | 0.03 | 97.17 | 91.97 |
| SO_U1_down | 41740262 | 40293836 | 6.04 G | 0.03 | 97.16 | 91.92 |
| SO_U2_up | 45784884 | 43954448 | 6.59 G | 0.03 | 97.02 | 91.64 |
| SO_U2_down | 41700460 | 40180500 | 6.03 G | 0.03 | 97.14 | 91.92 |
| SO_U3_up | 41575614 | 40393868 | 6.06 G | 0.03 | 97.21 | 92.03 |
| SO_U3_down | 46997140 | 43881100 | 6.58 G | 0.03 | 97.32 | 92.29 |
| ‘Orah’_D1_up | 46457488 | 43499296 | 6.52 G | 0.03 | 97.31 | 92.45 |
| ‘Orah’_D1_down | 47955910 | 45738074 | 6.86 G | 0.03 | 97.25 | 92.32 |
| ‘Orah’_D2_up | 43059442 | 40059634 | 6.01 G | 0.02 | 98.05 | 94.31 |
| ‘Orah’_D2_down | 45705752 | 41577146 | 6.24 G | 0.03 | 97.88 | 93.90 |
| ‘Orah’_D3_up | 50805306 | 48081564 | 7.21 G | 0.03 | 97.24 | 92.32 |
| ‘Orah’_D3_down | 42489004 | 41758214 | 6.26 G | 0.03 | 97.90 | 93.92 |
| ‘Orah’_U1_up | 43819358 | 41491262 | 6.22 G | 0.03 | 97.56 | 92.97 |
| ‘Orah’_U1_down | 46746712 | 43787796 | 6.57 G | 0.03 | 97.22 | 92.11 |
| ‘Orah’_U2_up | 46613288 | 43623750 | 6.54 G | 0.03 | 97.21 | 92.11 |
| ‘Orah’_U2_down | 43738976 | 41415628 | 6.21 G | 0.03 | 97.38 | 92.63 |
| ‘Orah’_U3_up | 41958940 | 39749244 | 5.96 G | 0.03 | 97.32 | 92.45 |
| ‘Orah’_U3_down | 46775178 | 44644718 | 6.70 G | 0.02 | 98.24 | 94.61 |

Note: SO: Sweet orange (*Citrus sinensis* (L.) Osbeck); D: Huanglongbing-affected; U: Mineral nutrient-deficiency-affected; Up: Younger mature (upper) leaves; Down: Older mature (lower) leaves. E.g., 'SO_D_up' denotes the younger mature leaves of sweet orange shoots infected with CLas.

Table S5 Summary of genome mapping results using RNA-seq data of each sample

| **Sample** | **Total_reads** | **Total_map** | **Unique_map** |
| --- | --- | --- | --- |
| SO_D1_up | 43213526 | 40387903 (93.46%) | 38545689 (89.20%) |
| SO_D1_down | 45019976 | 41741704 (92.72%) | 39857206 (88.53%) |
| SO_D2_up | 40838436 | 37833205 (92.64%) | 36094197 (88.38%) |
| SO_D2_down | 44939234 | 41637032 (92.65%) | 39783340 (88.53%) |
| SO_D3_up | 40327634 | 37469074 (92.91%) | 35768057 (88.69%) |
| SO_D3_down | 39517972 | 36715325 (92.91%) | 35049782 (88.69%) |
| SO_U1_up | 40716514 | 37853537 (92.97%) | 35901639 (88.17%) |
| SO_U1_down | 40293836 | 37528880 (93.14%) | 35713582 (88.63%) |
| SO_U2_up | 43954448 | 40875390 (92.99%) | 38971413 (88.66%) |
| SO_U2_down | 40180500 | 37462273 (93.23%) | 35698354 (88.84%) |
| SO_U3_up | 40393868 | 37757196 (93.47%) | 35968305 (89.04%) |
| SO_U3_down | 43881100 | 40685921 (92.72%) | 38765980 (88.34%) |
| ‘Orah’_D1_up | 43499296 | 39861653 (91.64%) | 38071106 (87.52%) |
| ‘Orah’_D1_down | 45738074 | 40983685 (89.61%) | 38833285 (84.9%) |
| ‘Orah’_D2_up | 40059634 | 36742339 (91.72%) | 34969579 (87.29%) |
| ‘Orah’_D2_down | 41577146 | 37835274 (91.00%) | 36035468 (86.67%) |
| ‘Orah’_D3_up | 48081564 | 43776613 (91.05%) | 41693483 (86.71%) |
| ‘Orah’_D3_down | 41758214 | 38516377 (92.24%) | 36670679 (87.82%) |
| ‘Orah’_U1_up | 41491262 | 37912858 (91.38%) | 35982799 (86.72%) |
| ‘Orah’_U1_down | 43787796 | 39244997 (89.63%) | 37152158 (84.85%) |
| ‘Orah’_U2_up | 43623750 | 39726482 (91.07%) | 37775206 (86.59%) |
| ‘Orah’_U2_down | 41415628 | 37553381 (90.67%) | 35712552 (86.23%) |
| ‘Orah’_U3_up | 39749244 | 36313311 (91.36%) | 34515946 (86.83%) |
| ‘Orah’_U3_down | 44644718 | 40561280 (90.85%) | 38321827 (85.84%) |

Note: SO: Sweet orange (*Citrus sinensis* (L.) Osbeck); D: Huanglongbing-affected; U: Mineral nutrient-deficiency-affected; Up: Younger mature (upper) leaves; Down: Older mature (lower) leaves. E.g., 'SO_D_up' denotes the younger mature leaves of sweet orange shoots infected with CLas.

Table S6 The DEGs related to phenylpropanoid biosynthesis and brassinosteroid biosynthesis pathway in Group 1 and Group 2

| **Pathway** | **Gene_id** | **log_2_Fold change** | | **Gene_description** |
| --- | --- | --- | --- | --- |
|  |  | **Group 1** | **Group 2** |  |
| Phenylpropanoid biosynthesis | 102623419 | 2.71 | 0.00 | 4-coumarate--CoA ligase 1-like |
|  | 102631295 | -1.57 | 0.00 | Anthocyanidin 3-O-glucosyltransferase 5-like |
|  | 102627553 | -1.51 | 0.00 | Anthranilate N-methyltransferase-like |
|  | 102620533 | -1.40 | 0.00 | Anthranilate N-methyltransferase-like |
|  | 102630049 | 2.48 | 0.00 | Anthranilate N-methyltransferase-like |
|  | 102610871 | 0.00 | 2.00 | Anthranilate N-methyltransferase-like |
|  | 102609976 | 1.46 | 1.55 | Anthranilate N-methyltransferase-like transcript variant X1 |
|  | 102611981 | 0.00 | 1.01 | Anthranilate N-methyltransferase-like transcript variant X1 |
|  | 102624507 | 1.94 | 1.82 | Beta-glucosidase 11-like |
|  | 102607393 | 5.19 | 4.10 | Beta-glucosidase 46-like |
|  | 102629858 | 1.76 | 0.00 | Caffeic acid 3-O-methyltransferase |
|  | novel.907 | 0.00 | 1.43 | Caffeic acid 3-O-methyltransferase |
|  | 102612776 | 0.00 | 1.54 | Caffeic acid 3-O-methyltransferase 1-like transcript variant X1 |
|  | 102613962 | 0.00 | 2.42 | Caffeic acid 3-O-methyltransferase 1-like transcript variant X2 |
|  | 102609652 | 0.00 | 1.37 | Caffeic acid 3-O-methyltransferase-like |
|  | 102620276 | 0.00 | -4.31 | Caffeic acid 3-O-methyltransferase-like |
|  | 102617232 | 0.00 | -1.13 | Caffeoyl-CoA O-methyltransferase-like |
|  | 102615114 | 1.87 | 1.41 | Cinnamoyl-CoA reductase 1-like transcript variant X1 |
|  | 102613413 | 0.00 | -3.48 | Feruloyl CoA ortho-hydroxylase 1 |
|  | 102612308 | 0.00 | -3.77 | Feruloyl CoA ortho-hydroxylase 1 transcript variant X2 |
|  | 102610999 | -3.33 | 0.00 | Peroxidase 10 transcript variant X2 |
|  | 102624858 | -3.42 | -2.90 | Peroxidase 12 |
|  | 102613627 | 0.00 | -1.53 | Peroxidase 15-like |
|  | 102614517 | 0.00 | -1.36 | Peroxidase 15-like |
|  | 102615475 | 0.00 | -2.75 | Peroxidase 15-like |
|  | 102622610 | -3.77 | -2.72 | Peroxidase 17 |
|  | 102629002 | 0.00 | 2.09 | Peroxidase 25 transcript variant X1 |
|  | 102617251 | 2.98 | 4.02 | Peroxidase 3 |
|  | 102630558 | 2.31 | 1.36 | Peroxidase 31-like |
|  | 102631301 | 0.00 | 1.61 | Peroxidase 42 |
|  | 102611617 | 2.04 | 1.14 | Peroxidase 47 |
|  | 102630988 | 1.71 | 1.93 | Peroxidase 4-like |
|  | 102625757 | 0.00 | -2.08 | Peroxidase 4-like |
|  | 102614803 | 0.00 | -2.46 | Peroxidase A2 |
|  | 102620173 | 2.38 | 0.00 | Phenylalanine ammonia-lyase |
|  | 102615640 | 3.03 | 0.00 | Scopoletin glucosyltransferase |
|  | 102615067 | 1.80 | 0.00 | Scopoletin glucosyltransferase-like |
|  | 102628720 | -3.13 | -2.36 | Shikimate O-hydroxycinnamoyltransferase-like |
|  | 102613844 | 1.12 | 0.00 | Shikimate O-hydroxycinnamoyltransferase-like |
|  | 102617597 | 1.09 | 1.13 | Shikimate O-hydroxycinnamoyltransferase-like |
|  | 102609895 | 0.00 | 5.09 | Shikimate O-hydroxycinnamoyltransferase-like |
|  | 102606992 | 0.00 | -1.20 | UDP-glycosyltransferase 72E1-like |
|  | 102629109 | 0.00 | 1.40 | Vicianin hydrolase-like |
|  | 102625849 | -1.62 | 0.00 | Vinorine synthase-like |
|  | 107177502 | 0.00 | -4.04 | Vinorine synthase-like |
| Brassinosteroid biosynthesis | 102622791 | 0.00 | -1.33 | Cytochrome P450 71A1-like |
|  | 102608450 | 0.00 | -1.76 | Cytochrome P450 90A1-like |
|  | 102623486 | 0.00 | -1.22 | Cytochrome P450 90B1 transcript variant X1 |
|  | 102623755 | 0.00 | -2.30 | Cytochrome P450 CYP749A22-like |
|  | 102622879 | 0.00 | -3.21 | Cytochrome P450 CYP749A22-like transcript variant X1 |
|  | 102610008 | -1.28 | 0.00 | 3-epi-6-deoxocathasterone 23-monooxygenase transcript variant X2 |
|  | 102623590 | 0.00 | 0.00 | 3-epi-6-deoxocathasterone 23-monooxygenase transcript variant X2 |
|  | 112496031 | 1.78 | 0.00 | - |

Table S7 The DEGs related to photosynthesis in the Zinc-deficiency-like younger leaves than in the blotchy mottle mature leabes of Huanglongbing-affected sweet orange

| **KEGG_pathway** | **Gene_id** | **log_2_Fold change** | **Gene_description** |
| --- | --- | --- | --- |
| Photosynthesis | 102626023 | 2.01 | Photosystem I subunit O |
|  | 102622651 | 1.82 | Plastocyanin |
|  | 102623297 | 1.70 | Photosystem I reaction center subunit psaK chloroplastic |
|  | 102615667 | 1.62 | Photosystem II repair protein PSB27-H1 chloroplastic |
|  | 102609364 | 1.59 | Ferredoxin leaf L-A-like |
|  | 102620317 | 1.56 | Oxygen-evolving enhancer protein 3-2 chloroplastic |
|  | 102629478 | 1.51 | Photosystem II reaction center W protein chloroplastic |
|  | 102610209 | 1.51 | Photosystem II reaction center W protein chloroplastic-like |
|  | 102622955 | 1.45 | Photosystem I reaction center subunit Vchloroplastic |
|  | 4271117 | 1.43 | - |
|  | 102630024 | 1.39 | Photosystem II 22 kDa protein chloroplastic |
|  | 102621051 | 1.38 | Oxygen-evolving enhancer protein 1 chloroplastic |
|  | 102616527 | 1.38 | Photosystem I reaction center subunit VI-2 chloroplastic |
|  | 102630675 | 1.35 | Photosystem II core complex proteins psbY chloroplastic |
|  | 102578062 | 1.31 | PSI reaction center subunit II |
|  | 102610576 | 1.31 | Photosynthetic NDH subunit of lumenal location 3 chloroplastic |
|  | 102611607 | 1.28 | Photosystem I reaction center subunit N chloroplastic |
|  | 102608346 | 1.24 | Photosynthetic NDH subunit of lumenal location 2 chloroplastic |
|  | 102625749 | 1.21 | Oxygen-evolving enhancer protein 2 chloroplastic |
|  | 102577948 | 1.19 | PSI reaction center subunit III |
|  | 102606718 | 1.16 | Cytochrome b6-f complex iron-sulfur subunitchloroplastic |
|  | 102629991 | 1.12 | ATP synthase gamma chain chloroplastic |
|  | 102577962 | 1.11 | PSI reaction center subunit II |
|  | 102621724 | 1.10 | Ferredoxin--NADP reductase leaf-type isozyme Chloroplastic transcript variant X2 |
|  | 102630095 | 1.04 | Ferredoxin C 1 chloroplastic |
|  | 102616249 | 1.00 | Photosystem I reaction center subunit III chloroplastic |
|  | 102610762 | 0.90 | ATP synthase subunit delta chloroplastic |
|  | 102609622 | 0.88 | Photosystem I reaction center subunit XI chloroplastic |
|  | 102629780 | 0.85 | PsbP-like protein 1 chloroplastic |
|  | 102611091 | 0.79 | Photosystem II reaction center PSB28 protein chloroplastic transcript variant X1 |
|  | 102618936 | 0.69 | Ferredoxin C 2 chloroplastic |
|  | 102608949 | -0.81 | Ferredoxin root R-B2 |
|  | 102612085 | -0.94 | Ferredoxin root R-B1-like transcript variant X1 |
| Photosynthesis - antenna proteins | 102625853 | 2.02 | Chlorophyll a-b binding protein of LHCII type 1 |
|  | 102609195 | 1.89 | Chlorophyll a-b binding protein CP26 chloroplastic |
|  | 102625194 | 1.81 | Chlorophyll a-b binding protein 13 chloroplastic |
|  | 102609463 | 1.69 | Chlorophyll a-b binding protein CP24 10A chloroplastic |
|  | 102610533 | 1.64 | Chlorophyll a-b binding protein 151 chloroplastic |
|  | 102613923 | 1.60 | Chlorophyll a-b binding protein 8 chloroplastic |
|  | 102629975 | 1.50 | Chlorophyll a-b binding protein 6 chloroplastic |
|  | 102630539 | 1.50 | Chlorophyll a-b binding protein P4 chloroplastic |
|  | 102625299 | 1.36 | Chlorophyll a-b binding protein of LHCII type 1 |
|  | 102608150 | 1.30 | Chlorophyll a-b binding protein chloroplastic |
|  | 102626435 | 1.23 | Chlorophyll a-b binding protein of LHCII type 1 transcript variant X2 |
|  | 102627462 | 1.18 | Chlorophyll a-b binding protein CP29.1 chloroplastic |
|  | 102613058 | 0.93 | Photosystem I chlorophyll a/b-binding protein 5 chloroplastic transcript variant X3 |
|  | 102607051 | 0.85 | Photosystem I chlorophyll a/b-binding protein 6chloroplastic transcript variant X1 |
|  | 102612622 | 0.79 | Chlorophyll a-b binding protein 7 chloroplastic transcript variant X1 |
|  | 102577995 | 0.78 | Chlorophyll a-b binding protein CP29.3 chloroplastic |
| Carotenoid biosynthesis | 102611561 | 1.28 | Abscisic acid 8'-hydroxylase 3-like |
|  | 102577994 | 1.25 | Violaxanthin de-epoxidase |
|  | 102612995 | 0.88 | Probable carotenoid cleavage dioxygenase 4 chloroplastic |
|  | 102615387 | 0.82 | Zeta-carotene desaturase chloroplastic/chromoplastic-like |
|  | 102577984 | 0.71 | Zeaxanthin epoxidase chloroplastic transcript variant X1 |
|  | 102578001 | 0.64 | Phytoene synthase transcript variant X1 |
|  | 102577969 | 0.64 | ZDS |
|  | 102625209 | -0.96 | Capsanthin/capsorubin synthase chromoplastic-like |
|  | 102578064 | -1.12 | Putative 9-cis-epoxycarotenoid dioxygenase 3 |
|  | 102578000 | -1.57 | ABA glucosyltransferase |
| Porphyrin and chlorophyll metabolism | 102616348 | 1.69 | Magnesium-chelatase subunit ChlH chloroplastic |
|  | 102612985 | 1.55 | Magnesium-protoporphyrin IX monomethyl ester [oxidative] cyclase chloroplastic |
|  | 102614959 | 1.54 | Protochlorophyllide reductase chloroplastic transcript variant X2 |
|  | 102607042 | 1.37 | Protein STAY-GREEN LIKE chloroplastic transcript variant X2 |
|  | 102628832 | 1.21 | Geranylgeranyl diphosphate reductase chloroplastic |
|  | 102606628 | 0.98 | Chlorophyllide a oxygenase chloroplastic |
|  | 102629883 | 0.87 | Magnesium protoporphyrin IX methyltransferase chloroplastic |
|  | 102630676 | 0.81 | Porphobilinogen deaminase chloroplastic |
|  | 102620862 | 0.75 | Magnesium-chelatase subunit ChlI chloroplastic |
|  | 102611623 | 0.74 | Divinyl chlorophyllide a 8-vinyl-reductase chloroplastic |
|  | 102624855 | 0.63 | 7-hydroxymethyl chlorophyll a reductase chloroplastic transcript variant X3 |
|  | 102624392 | 0.60 | Glutamyl-tRNA reductase 1 chloroplastic |
|  | 102625350 | -1.19 | Protein STAY-GREEN chloroplastic |
|  | 102578008 | -1.43 | Chloroplast chlorophyllase |

Table S8 The DEGs related to carbon metabolism in Group 5

| **KEGG_pathway** | **Gene_id** | **log_2_Fold change** | **Gene_description** |
| --- | --- | --- | --- |
| Carbon metabolism | 102621953 | 3.76 | Alcohol dehydrogenase-like 2 transcript variant X1 |
|  | 102628345 | 1.94 | Fructose-16-bisphosphatase chloroplastic |
|  | 112496032 | 1.91 | Uncharacterized LOC112496032 |
|  | 102611272 | 1.82 | Cysteine synthase-like transcript variant X3 |
|  | 102612988 | 1.74 | Ribulose bisphosphate carboxylase small chain chloroplastic-like |
|  | 102623813 | 1.56 | Phosphoenolpyruvate carboxykinase (ATP)-like |
|  | 102627653 | 1.55 | Glycine dehydrogenase (decarboxylating) mitochondrial |
|  | 102621511 | 1.52 | Sedoheptulose-17-bisphosphatase chloroplastic |
|  | 102614363 | 1.45 | Glycine cleavage system H protein mitochondrial |
|  | 102610141 | 1.30 | Serine--glyoxylate aminotransferase |
|  | 102612734 | 1.27 | Phosphoribulokinase chloroplastic |
|  | 102622701 | 1.16 | Serine hydroxymethyltransferase mitochondrial |
|  | 102606603 | 1.16 | Fructose-bisphosphate aldolase 1 chloroplastic |
|  | 102614495 | 1.14 | Glyceraldehyde-3-phosphate dehydrogenase A chloroplastic |
|  | 102618745 | 1.08 | Malate dehydrogenase mitochondrial-like |
|  | 102617863 | 1.06 | (S)-2-hydroxy-acid oxidase GLO1 |
|  | 102626680 | 1.02 | Glyceraldehyde-3-phosphate dehydrogenase GAPB chloroplastic |
|  | 102630329 | 0.99 | Alcohol dehydrogenase-like 7 transcript variant X2 |
|  | 102613631 | 0.99 | Cysteine synthase-like transcript variant X1 |
|  | 102622394 | 0.98 | Malate dehydrogenase glyoxysomal transcript variant X3 |
|  | 102616367 | 0.94 | - |
|  | 102609480 | 0.92 | Ribulose-phosphate 3-epimerase chloroplastic transcript variant X2 |
|  | 102611457 | 0.90 | Phosphoglycerate kinase chloroplastic |
|  | 102611899 | 0.87 | Glutamate--glyoxylate aminotransferase 2 transcript variant X1 |
|  | 102625481 | 0.86 | Glycerate dehydrogenase |
|  | 102622984 | 0.81 | Malate dehydrogenase [NADP] chloroplastic |
|  | 102611458 | 0.80 | Probable 6-phosphogluconolactonase 1 transcript variant X1 |
|  | 102577940 | 0.74 | NADP-dependent glyceraldehyde-3-phosphate dehydrogenase |
|  | 102611751 | 0.73 | Bifunctional L-3-cyanoalanine synthase/cysteine synthase D2-like transcript variant X1 |
|  | 102608472 | 0.73 | Probable ribose-5-phosphate isomerase 4 chloroplastic transcript variant X1 |
|  | 102631066 | 0.70 | Phosphoglycolate phosphatase 1B chloroplastic |
|  | 102627315 | 0.67 | Pyruvate phosphate dikinase chloroplastic transcript variant X1 |
|  | 102619194 | 0.67 | - |
|  | 102624724 | 0.64 | Aminomethyltransferase mitochondrial transcript variant X3 |
|  | 102613688 | -0.61 | Acyl-coenzyme A oxidase 2 peroxisomal |
|  | 102611485 | -0.66 | Uncharacterized LOC102611485 transcript variant X2 |
|  | 102622925 | -0.68 | Formate dehydrogenase mitochondrial |
|  | 102620014 | -0.78 | Pyruvate kinase isozyme A chloroplastic-like |
|  | 102610613 | -0.79 | Ribose-phosphate pyrophosphokinase 1 transcript variant X1 |
|  | 102629862 | -0.93 | Alcohol dehydrogenase class-3 |
|  | 102615829 | -1.95 | Gluconokinase |
| Carbon fixation in photosynthetic organisms | 102628345 | 1.94 | Fructose-16-bisphosphatase chloroplastic |
|  | 102612988 | 1.74 | Ribulose bisphosphate carboxylase small chain chloroplastic-like |
|  | 102623813 | 1.56 | Phosphoenolpyruvate carboxykinase (ATP)-like |
|  | 102621511 | 1.52 | Sedoheptulose-17-bisphosphatase chloroplastic |
|  | 102612734 | 1.27 | Phosphoribulokinase chloroplastic |
|  | 102606603 | 1.16 | Fructose-bisphosphate aldolase 1 chloroplastic |
|  | 102614495 | 1.14 | Glyceraldehyde-3-phosphate dehydrogenase A chloroplastic |
|  | 102618745 | 1.08 | Malate dehydrogenase mitochondrial-like |
|  | 102626680 | 1.02 | Glyceraldehyde-3-phosphate dehydrogenase GAPB chloroplastic |
|  | 102622394 | 0.98 | Malate dehydrogenase glyoxysomal transcript variant X3 |
|  | 102609480 | 0.92 | Ribulose-phosphate 3-epimerase chloroplastic transcript variant X2 |
|  | 102611457 | 0.90 | Phosphoglycerate kinase chloroplastic |
|  | 102611899 | 0.87 | Glutamate--glyoxylate aminotransferase 2 transcript variant X1 |
|  | 102622984 | 0.81 | Malate dehydrogenase [NADP] chloroplastic |
|  | 102608472 | 0.73 | Probable ribose-5-phosphate isomerase 4 chloroplastic transcript variant X1 |
|  | 102627315 | 0.67 | Pyruvate phosphate dikinase chloroplastic transcript variant X1 |
|  | 102611485 | -0.66 | Uncharacterized LOC102611485 transcript variant X2 |

Figure S1 Analysis of correlation between samples

Note: SO: Sweet orange (*Citrus sinensis* (L.) Osbeck); D: Huanglongbing-affected; U: Mineral nutrient-deficiency-affected; Up: Younger mature (upper) leaves; Down: Older mature (lower) leaves. E.g., 'SO_D_up' denotes the younger mature leaves of sweet orange shoots infected with CLas.

Figure S2 Principal component analysis (PCA) plots for RNA-seq data of samples

Note: SO: Sweet orange (*Citrus sinensis* (L.) Osbeck); D: Huanglongbing-affected; U: Mineral nutrient-deficiency-affected; Up: Younger mature (upper) leaves; Down: Older mature (lower) leaves. E.g., 'SO_D_up' denotes the younger mature leaves of sweet orange shoots infected with CLas.

Figure S3 Log_2_Fold change cluster diagram of the DEGs in the photosynthesis pathway

Note: Group 1, SO_D_up *vs.* SO_U_up; Group 2, ‘Orah’_D_up *vs.* ‘Orah’_U_up.
